# 6-PPD induces mitochondrial dysfunction and reduces healthspan and lifespan through SKN-1 in *Caenorhabditis elegans*

**DOI:** 10.1016/j.jhazmat.2025.140332

**Published:** 2025-10-31

**Authors:** Moonjung Hyun, Laxmi Rathor, Hye-Jin Kim, Ho-Jeong Lee, Taylor McElroy, Moongi Ji, Min-Seok Choi, Steven Beck, Julia Bailey, Hun Hwan Kim, Yi Sheng, Jeong-Doo Heo, Stephanie Wohlgemuth, Rui Xiao, Gon Sup Kim, Man-Jeong Paik, Yong-Hyun Kim, Sung-Hwan Kim, Myon Hee Lee, Sung Min Han

**Affiliations:** a Division of Gyeongnam Bio-Environmental Research, Bio-Health Research Center, Jinju 52834, Republic of Korea; b Department of Physiology and Aging, College of Medicine, Institute on Aging, University of Florida, Gainesville, FL 32610, USA; c College of Pharmacy, Sunchon National University, Suncheon 57922, Republic of Korea; d Department of Environmental Engineering, Jeonbuk National University, Jeonju 54896, Republic of Korea; e College of Veterinary Medicine, Gyeongsang National University, Jinju 52828, Republic of Korea; f Division of Jeonbuk Advanced Bio Research, Korea Institute of Toxicology, Jeongeup 56212, Republic of Korea; g Department of Internal Medicine, Hematology/Oncology Division, Brody School of Medicine at East Carolina University, Greenville, NC 27834, USA; h Department of Biology, East Carolina University, Greenville, NC 27858, USA

**Keywords:** 6-PPD, 6-PPDQ, Healthspan, Lifespan, Mitochondria, SKN-1/Nrf2, MEF, *C. elegans*

## Abstract

The synthetic antioxidant N-(1,3-dimethylbutyl)-N’-phenyl-p-phenylenediamine (6-PPD) is widely used in rubber products, but its toxicity in non-aquatic animals remains poorly understood. We investigated 6-PPD toxicity in mouse embryonic fibroblasts (MEFs) and *Caenorhabditis elegans* (*C. elegans*). In MEFs, 6-PPD caused dose-dependent increases in cell death, apoptosis, and mitochondrial dysfunction, along with elevated reactive oxygen species (ROS). In *C. elegans*, 6-PPD exposure led to higher mortality, delayed development, reduced reproduction, and shortened lifespan. Mitochondrial impairment, increased ROS, decreased mobility, and weakened stress tolerance were also observed. High-performance liquid chromatography (HPLC) analysis detected trace amounts of 6-PPD in *C. elegans*, whereas 6-PPD Quinone (6-PPDQ) was not detected within the analytical range. Furthermore, we found that 6-PPD upregulated the conserved transcription factor skinhead-1 (SKN-1/Nrf2) transcription factor. While SKN-1 was necessary for the reduced lifespan and early mortality caused by 6-PPD exposure, its loss failed to prevent the mitochondrial defects induced by 6-PPD exposure. Notably, SKN-1 gain-of-function mutations under basal conditions impaired mitochondrial function, and 6-PPD exposure further exacerbated these defects. These findings indicate that 6-PPD disrupts mitochondria through both SKN-1-dependent and SKN-1-independent mechanisms. Overall, our study demonstrates that 6-PPD impairs development, reproduction, healthspan, and lifespan through the SKN-1/Nrf2 pathway, with mitochondrial dysfunction mediated by both SKN-1/Nrf2-dependent and independent mechanisms

## GRAPHICAL ABSTRACT

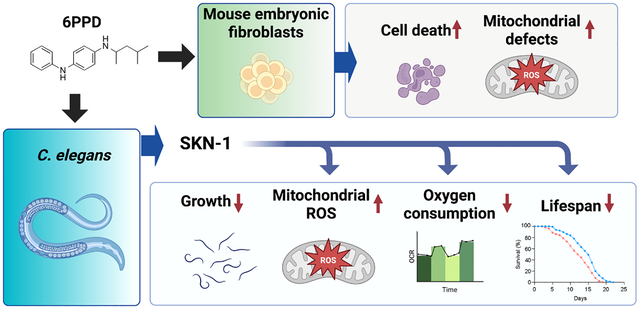

## 1. Introduction

The widespread use of synthetic chemicals across various industries has raised significant concerns for environmental and public health [52]. Chemicals employed in the rubber and tire industry have drawn particular attention due to their environmental persistence and potential toxicity. One such chemical, N-(1,3-Dimethylbutyl)-N’-phenyl-p--phenylenediamine (6-PPD), is widely used as an antioxidant to protect rubber materials from cracking or wear-out caused by environmental factors, such as heat, oxygen, and ozone [5,42,48]. It undergoes a chemical transformation in the environment, reacting with ozone to form 6-PPD Quinone (6-PPDQ), an emerging contaminant of significant concern [93]. Both 6-PPD and its transformation product, 6-PPDQ, have been detected in various environmental media, including stormwater, snowmelt, surface water, and wastewater across different climatic regions [12,15,50,51,83,87]. However, the concentrations of these compounds vary by medium. For example, roadside soils and road dust, particularly in urban areas such as Guangzhou, Tokyo, and Hong Kong, contain high levels of 6-PPD [11,19,33,34,61]. In air particles, 6-PPD concentrations range from 0.02 to 9340 pg/m^3^, while 6-PPDQ levels range from 0.1 to 84 pg/m^3^, exhibiting regional variability [11,99,110, 111]. In runoff and surface waters, 6-PPD concentrations reach up to 2710 ng/L, while 6-PPDQ levels reach 2430 ng/L, with stormwater and wastewater treatment plants also showing detectable levels of these compounds [11,50,51,83,87]. Thus, 6-PPD is prevalent in both dust and road-associated matrices due to direct deposition from TWP, whereas 6-PPDQ dominates aqueous environments, likely due to its oxidative transformation [11,19,33,34,61,87].

Their widespread presence raises concerns about potential adverse effects on human health. Recent studies in China have detected both compounds in human urine, with detection frequencies ranging from 60 % to 100 %. Notably, pregnant women exhibited higher concentrations of both compounds (median: 0.068 ng/mL for 6-PPD and 2.91 ng/mL for 6-PPDQ) compared to adults (0.018 ng/mL and 0.40 ng/mL, respectively) and children (0.015 ng/mL and 0.076 ng/mL, respectively) [24]. In a study of human serum samples from China, both 6-PPD and 6-PPDQ were frequently detected, with a median concentration of 0.057 ng/mL and 0.13 ng/mL, respectively [88]. In another study, although 6-PPD levels were not measured, 6-PPDQ was detected in 100 % of urine samples from 15 healthy adults in China, with a median concentration of 0.89 ng/mL [21]. Furthermore, in the cerebrospinal fluid (CSF) of Parkinson’s disease patients, 6-PPDQ was detected at a concentration of 11.18 ng/mL, which was higher than in healthy individuals (5.087 ng/mL) [27]. Collectively, these findings highlight the potential for human exposure to 6-PPD and 6-PPDQ under various conditions and suggest a possible association with human health.

Growing evidence suggests that 6-PPD is a potent environmental contaminant with diverse toxic effects across taxa. In zebrafish, exposure to 6-PPD induces oxidative stress, apoptosis, mitochondrial damage, impaired autophagic degradation, cardiac dysfunction, and myocardial injury [25,26]. Other studies on other aquatic vertebrates, including amphibians, report that 6-PPD exposure disrupts lipid metabolism, impairs development, inhibits growth, and suppresses immune function, increasing susceptibility to environmental stressors [65]. Although research on mammals is limited recent research shows that both 6-PPD and its oxidized derivative 6-PPDQ can promote the proliferation and migration of non-small cell lung cancer cells by disrupting tumor suppressor PTEN function and reprogramming cellular metabolism, suggesting a potential role in cancer progression and highlighting further health risks [97]. Extensive research has focused on the toxicity of 6-PPDQ due to recent findings that exposure to this 6-PPD derivative from urban runoff causes acute mortality in coho salmon (Oncorhynchus kisutch), drawing significant attention [93]. Beyond acute lethality, 6-PPDQ has been shown to impair reproduction, induce developmental abnormalities, and cause neurotoxicity in various marine and freshwater fish models [25,26,34,93,95,96,109]. However, different species exhibit varying sensitivities to 6-PPDQ, with some species being more susceptible than others [25,26,95,109]. In mice, exposure to 6-PPDQ induces oxidative stress, inflammation, and neurotoxicity, leading to cognitive deficits, reduced motor coordination, decreased sperm quality, and impaired reproductive capacity [70,107]. Thus, although most studies have focused on 6-PPDQ exposure, the direct effects of 6-PPD exposure are not yet well understood.

*C. elegans* is a well-established model organism in environmental toxicology, known for its ease of maintenance, genetic tractability, short lifespan, fully described developmental program, and conserved molecular pathways related to development, metabolism, and stress responses [46,47,60]. Previous studies have leveraged these advantages to characterize the long-term toxicity of 6-PPDQ, including disruptions in lipid and glucose metabolism, increased intestinal permeability, and elevated reactive oxygen species (ROS) production [66,101,102]. Additionally, 6-PPDQ exposure has been associated with impaired mitochondrial function, increased germline apoptosis, reproductive impairments, neurotoxicity, and accelerated aging [36–38,40,41,43,45, 64,67,103]. However, the toxicity and underlying mechanisms of 6-PPD remain poorly understood. Given the detection of both 6-PPD and 6-PPDQ in humans and their species-specific toxicological effects, it is crucial to investigate both compounds further to better understand their potential risks and biological impacts.

In this study, we investigated the toxicological effects of 6-PPD using both mammalian cell models and *C. elegans*. Our results show that 6-PPD exposure caused significant mitochondrial dysfunction, increased apoptosis, and elevated mortality in mouse embryonic fibroblasts (MEFs). In *C. elegans*, 6-PPD exposure led to increased mortality, delayed development, reduced reproductive capacity, impaired healthspan, and a shortened lifespan. Both systems exhibited increased ROS production, highlighting the pivotal role of mitochondrial health in 6-PPD toxicity. Mechanistically, we found that the activation of the SKN-1/Nrf2 signaling pathway in response to 6-PPD exposure was required for reduced stress responses and affected longevity. This study offers valuable insights into the toxicological impact of 6-PPD, particularly its effects on mitochondrial function, development, aging, and conserved stress response mechanisms.

## 2. Materials and methods

### 2.1. Chemicals

6-PPD (Sigma, USA, #CDS013697) was used for MEF and *C. elegans* assays. 6-PPD (TCI, Japan, #D3331) was used for C. *elegans* HPLC-UV analysis. 6-PPDQ (HPC Inc., Germany, #687885) and Dimethyl sulfoxide (Sigma, USA, #D2650, 99.7 % pure) were also employed in the study.

### 2.2. Cell culture

Wild-type (WT) MEF cells were provided by Dr. Andrew Larner (Virginia Commonwealth University). The cells were routinely cultured in Dulbecco’s Modified Eagle’s Medium without sodium pyruvate, supplemented with 10 % heat-inactivated fetal bovine serum (ThermoFisher Scientific, USA, #10082147) and penicillin-streptomycin (10,000 U/mL; ThermoFisher Scientific, USA, Cat #15140122). The cultured cells were exposed to increasing concentrations of 6-PPD/6-PPDQ, ranging from 10 μM to 50 μM. Control plates were prepared by adding 0.5 % DMSO alone and represented by 0 μM as a 6-PPD or 6-PPDQ untreated condition.

### 2.3. Cell viability

WT MEF cells (2 × 10^5^ cells/mL) were seeded in six-well plates. After 48 h, the cells were exposed to 6-PPD/6-PPDQ, its three substitutes, or 0.5 % DMSO alone for 24 h. At the end of treatment, the medium was removed, and MTT (3-(4,5-dimethylthiazol-2-yl)-2,5-diphenyl-2H-tetrazolium bromide) (0.5 mg/mL, dissolved in PBS) was added to each well for 1 h. The color intensity was measured at 540 nm using a microplate reader (Synergy HTX, BioTek, USA).

### 2.4. Mitochondrial ROS level measurement in MEFs

WT MEF cells (2 × 10^5^ cells/mL) were seeded in six-well plates. After 48 h, the cells were exposed to either 0.5 % DMSO (control) or 6-PPD/6-PPDQ for 24 h. Mitochondrial ROS levels were measured by staining the cells with 5 μM MitoSOX Red (Invitrogen, USA, #M36008) at 37 °C for 20 min. After staining, the cells were washed and analyzed by flow cytometry (CytoFLEX, Beckman Coulter, USA). At least three independent experiments were performed for each condition.

### 2.5. Measurement of mitochondrial membrane potential in MEFs

WT MEF cells (2 × 10^5^ cells/mL) were seeded in 6-well plates and treated with 6-PPD/6-PPDQ for 24 h. After treatment, the mitochondrial membrane potential (Δψm) was assessed using the TMRE Assay Kit (Abcam, UK, #ab113852). The cells were incubated with 1 μM TMRE at 37 °C for 30 min and then washed twice with 1x PBS/2 % BSA. After washing, fluorescence intensity was detected using a microplate reader (SYNERGY HTX, BioTek, USA).

### 2.6. Apoptosis assay in MEFs

Apoptotic cells were detected using an apoptosis detection kit (ThermoFisher Scientific, USA, #V13242) by flow cytometry. A total of 2 × 10^5^ cells were seeded in 6-well plates. After 48 h, the cells were treated with either 0.5 % DMSO or 6-PPD/6-PPDQ for 24 h at 37 °C. Following the treatment, the cells were washed twice with warm 1x PBS and stained with FITC/PI using an apoptosis kit according to the manufacturer’s instructions. After staining, the cells were washed and analyzed using flow cytometry (CytoFLEX, Beckman Coulter, USA).

### 2.7. SDS-PAGE and Western blotting

WT MEF cells (2 × 10^5^ MEF cells/mL) were seeded in 6-well plates. After 48 h, the cells were treated with either 0.5 % DMSO or 6-PPD/6-PPDQ for 24 h at 37 °C. Following the treatment, the cells were washed twice with ice-cold 1x PBS and then resuspended in RIPA lysis buffer (Pierce, # 89900). Equal amounts of protein were then loaded onto Tris-glycine gels and subjected to SDS-PAGE. After electrophoresis, the proteins were transferred to polyvinylidene difluoride membranes using an iBlot 3 Gel Transfer Device (Invitrogen). The membrane was blocked for 1 h in 5 % non-fat milk or 5 % BSA in 1 × Tris-buffered saline (TBS) with 0.1 % Tween 20, and incubated overnight at 4 °C with shaking with the following primary antibodies: Grim-19 (Abcam, #ab110240), SDHA (Invitrogen, #459200), Cytochrome c (Abcam, #ab110325), COX IV (Abcam, #ab33985), and β-actin (Sigma-Aldrich, #A5441). After incubation with secondary antibodies (1:5000 dilution) for 1 h at room temperature in 5 % milk in 1 × TBS with 0.1 % Tween 20, bands were visualized using ECL solution (GE Healthcare, RPN2106, Thermo Fisher Scientific, #80196) and calibrated using a Chemi-Doc Imaging System (Bio-Rad, Hercules, CA, USA). Densitometric quantification was performed using ImageJ software (NIH, Bethesda, MD, USA).

### 2.8. C. elegans maintenance, strains, and 6-PPD treatment

The following strains were maintained at 20 °C by standard methods, unless otherwise stated, on nematode growth medium (NGM) agar plates seeded with the OP50 strain of *Escherichia coli* (*E. coli*), as previously described [8]: WT N2 (Bristol Laboratories), QV225 *skn-1(zj15) IV*, CL2166 *dvIs19 [(pAF15)gst-4p::GFP::NLS] III*, TJ356 *zIs356 [daf-16p:: daf-16a/b::GFP* + *rol-6(su1006)] IV*, SPC167 *dvIs19 [(pAF15) gst-4p:: GFP::NLS] III*, HAN474 *skn-1(lax188) IV*, and LD7 *ldIs7 [skn-1b/c::GFP* + *rol-6(su1006)]*. N2, mutant, and transgenic strains used in this study were provided by the Caenorhabditis Genetic Center (CGC) or Dr. Keith Blackwell’s lab (Harvard University). For 6-PPD treatment, a 6-PPD stock solution was prepared at a concentration of 250 mM in 100 % DMSO and stored at 4 °C. Chemical plates were prepared by adding 6-PPD to the NGM media at final concentrations ranging from 0.1 mM to 0.5 mM. Control plates were prepared by adding DMSO alone at the same concentration (0.2 % mM, v/v) and were labeled as 0 mM to indicate a 6-PPD-untreated condition.

### 2.9. C. elegans development rate assays

Three WT N2 larvae at the L4 stage (P0) were transferred to NGM plates containing 0, 0.1, or 0.5 mM 6-PPD. After 24 h, the P0 worms were removed, and age-synchronized F1 embryos were cultured continuously. The developmental stages of the F1 worms were evaluated after 3 days. A minimum of 350 worms were tested for developmental stages in each group. All experiments were performed in triplicate at 20 °C.

### 2.10. C. elegans reproduction assay

Five WT N2 larvae at the L4 stage (P0) were picked from normal NGM plates and transferred to NGM plates containing DMSO or 6-PPD. The P0 worms were transferred to fresh NGM plates containing DMSO or 6-PPD daily, and the number of laid embryos was scored under a microscope. The experiment was conducted until no eggs were laid. At least five independent experiments were performed for each condition to ensure the reliability of the results.

### 2.11. C. elegans lifespan assay

At least 20–30 age-synchronized L4 stage worms were transferred to NGM plates containing either DMSO or the indicated concentrations of 6-PPD. The age-synchronized L4-stage worms were transferred to fresh NGM plates containing either DMSO or 6-PPD every three days. Worm mortality was assessed daily by observing their response to a platinum wire touch. Individuals that failed to respond without movement were scored as dead, following standard protocols for determining mortality in *C. elegans* [17,78]. All experiments were conducted independently at least four times at 20 °C. To avoid artificial effects, we refrained from using fluorodeoxyuridine (FUdR), a DNA synthesis inhibitor typically used to block reproduction in the *C. elegans* lifespan assay [98].

### 2.12. C. elegans stress response assay

Age-synchronized L1 stage worms were cultured on NGM plates containing DMSO as a control or 6-PPD (0.1 mM or 0.5 mM) at 20 °C for approximately 72 h. For the oxidative stress analysis, synchronized 1-day-old adults were harvested from each condition and transferred to 1.5 mL microcentrifuge tubes (30–50 worms/tube) containing 0, 0.5, or 1 mM H_2_O_2_ or 100 mM paraquat in 1 mL M9 buffer. The worms were then incubated at 20 °C for 24 h. For the ultraviolet radiation (UV) stress assay, 20–30 synchronized L4-stage worms were transferred to fresh NGM plates containing either DMSO or 6-PPD and cultured until they reached the 1-day-old adult stage. The synchronized worms were then exposed to a UV dose of 1000 J/m^2^ in a UV chamber. For the viability assay, worms were considered dead if they did not respond to contact with a platinum wire after 48 h. At least three independent experiments were conducted for each condition.

### 2.13. C. elegans mobility assays

Behavior and body size assays were performed using an automated Worm Lab tracking system (MSCOP-010, MBF Bioscience, VT, USA) [3]. Age-synchronized WT L1 worms were placed on 6-PPD-containing NGM plates seeded with OP50 at 20 °C (30–50 worms/plate). When they reached the L4 stage (2 days after L1), worms were transferred again to fresh 6-PPD-containing NGM plates and cultured for 2 days at 20 °C. A single worm was then placed on a lightly spotted NGM plate not to induce foraging behavior, and its movement was recorded using the worm tracker instrument. The video was taken for 30 s using a Basler acA2500 camera, coupled with a 10x close-focus zoom lens to prevent intense light exposure during video recordings. Multiple behavioral parameters were tested, including body speed, body bending, body length, and body width. Body thrashing was tested on 2-day-old worms using a Pyrex watch glass and M9 buffer. Worms were left to acclimate in liquid for 1 min and recorded for 30 s. The thrashing rate was counted manually. At least three independent experiments were performed for each condition.

### 2.14. Mitochondrial staining in C. elegans

Tetramethylrhodamine Ethyl Ester Perchlorate (TMRE), MitoTracker Green FM, and MitoSOX Red were used to assess Δψm, mitochondrial mass, and mitochondrial ROS levels, respectively, in *C. elegans* [23,31]. For TMRE and MitoTracker Green FM staining, approximately 50 synchronized L4 larvae, pre-treated with DMSO or 6-PPD from the L1 stage, were transferred to fresh OP50-seeded NGM plates containing either 1 μM TMRE or 100 μM Green FM and incubated for two days at 20 °C. Afterward, worms were moved to regular NGM plates and incubated in the dark for ~1 h before being washed three times with M9 buffer to remove gut contents. For ROS detection, synchronized L1 worms were exposed to DMSO or 0.5 mM 6-PPD for 72 h at 20 °C. After exposure, worms were collected, washed, and resuspended in 500 μl of M9 buffer. Worms were then incubated with 10 μM MitoSOX^™^ Red at room temperature in the dark with gentle shaking for 1 h. After staining, worms were washed three times, transferred to fresh NGM plates without OP50, and incubated for an additional 5–10 min. Worms were then mounted onto 2 % agarose pads for imaging. Whole-body fluorescence intensity was measured using spinning-disk confocal microscopy (for TMRE and MitoTracker Green FM) or epifluorescence microscopy (for MitoSOX). At least three independent experiments were conducted for each condition.

### 2.15. Oxygen consumption rate in C. elegans

The oxygen consumption rate (OCR) was measured using a Seahorse XFe96 Analyzer (Agilent Technologies, CA, USA) following a protocol previously described with minor modifications [58,80]. Briefly, age-synchronized L1 stage WT N2 worms were cultured on NGM agar plates containing 6-PPD for 72 h and then transferred to fresh NGM plates containing the same concentration of 6-PPD. 2 days later, the worms were harvested and washed with M9 buffer at least three times. For each group, 10 worms/well were transferred to 96-well Seahorse XF96 V3 PS Cell Culture Microplates, with 10 technical replicates per treatment. Basal OCR was assessed in five 2-minute measuring cycles, with a 2-minute mixing period followed by a 30-second wait between measurements. Maximal uncoupled OCR was assessed after the injection of the uncoupler carbonyl cyanide-4 (trifluoromethoxy) phenylhydrazone (FCCP; 10 μM final concentration) in nine 2-minute measuring cycles, interrupted by mixing and waiting periods. Residual, non-mitochondrial OCR (ROX) was assessed after injecting sodium azide (40 mM final concentration), a complex-IV inhibitor, in four 2-minute measuring cycles, interrupted by mixing and waiting periods. The number of worms in each well was confirmed at the end of the assay with a Cytation-1 Imaging Multi-Mode Reader (BioTek Instruments, VT, USA). Data were acquired with the Agilent/Seahorse software Wave Desktop and Controller (v2.6.1) and analyzed with the Seahorse XF Cell Mito Stress Test Report Generator.

### 2.16. Measuring the absorbed concentration of 6-PPD/6-PPDQ in MEFs

6-PPD and 6-PPDQ analyses were performed using the LCMS-8050 system (Shimadzu, Kyoto, Japan). An ACQUITY UPLC HSS T3 column (2.1 mm × 150 mm, 1.8 μm) was used to separate 6-PPD and 6-PPDQ. The mass spectrometry (MS) parameters were set as follows: ionization mode: electrospray ionization mode interface temperature: 300 °C, desolvation line temperature: 250 °C, nebulizing gas flow: 3.0 L/min, heating gas flow: 10.0 L/min. The mobile phase was applied with gradient elution of 0.1 % formic acid in water and 0.1 % formic acid in acetonitrile. After 24-hour treatment with 6-PPD or 6-PPDQ, cells were harvested and lysed using an ultrasonicator to measure the intracellular 6-PPD and 6-PPDQ concentrations. Simultaneous analysis of 6-PPD and 6-PPDQ in WT MEF cells was performed by LC-MS/MS. Briefly, the deproteinization was performed by adding 100 μL of acetonitrile and 50 μg of the internal standard (3,4-dimethoxybenzoic acid) to lysed WT MEFs cells (1.0 ×10^4^) in an Eppendorf tube and mixing for 1 min. Subsequently, after centrifugation, the supernatant was filtered and transferred to an auto vial, which was then injected into the LC-MS/MS system.

### 2.17. Measuring the absorbed concentration of 6-PPD/6-PPDQ in C. elegans

For the quantification of 6-PPD and 6-PPDQ in *C. elegans*, the samples underwent pretreatment followed by analysis using an HPLC-UV system (Shimadzu, Kyoto, Japan). The *C. elegans* samples were homogenized using a tissue grinder and transferred into 2 mL Eppendorf tubes. Subsequently, 1 mL of acetonitrile (ACN) was added to each tube, and the mixture was vortexed at a speed of 3300 rpm for 1 min to facilitate the extraction of 6-PPD and 6-PPDQ into the solvent phase. Following extraction, the extract was centrifuged at 13,500 rpm for 10 s using a centrifuge (CF-12, DAIHAN Scientific, Republic of Korea) to separate particulates. The liquid sample was filtered through a hydrophobic polytetrafluoroethylene (PTFE) syringe filter (0.22 μm) to remove the solid particulates. The filtrates were then transferred into 2 mL amber vials and introduced into the HPLC-UV system via an automated sample injection unit. A 10 μL aliquot of each sample was injected for chromatographic analysis. The separation was performed using a C18 column (4.6 mm × 150 mm, 5 μm), with UV detection set at 290 nm to monitor the absorbance of 6-PPD and 6-PPDQ. The column oven temperature was maintained at 40 °C. The mobile phase consisted of a 4:6 (v/v) mixture of deionized water (DIW) and ACN, operating under isocratic elution conditions without buffer additives. The flow rate was maintained at 1.5 m/min. Quantitative determination of 6-PPD and 6-PPDQ was performed using external calibration curves constructed from liquid standard solutions. The calibration standards were prepared at five concentration levels, ranging from 49.0 ± 0.47 pg/μL to 979 ± 9.33 pg/μL, ensuring accurate quantification within the target concentration range.

### 2.18. Statistical analysis

For the comparisons of phenotypes between each concentration of 6-PPD treatment and control (DMSO-alone) at each time point, we analyzed the data using Prism software (version 10) (GraphPad Software, CA, USA). Unless indicated otherwise, the mean differences with a P value less than 0.05 were considered statistically significant.

## 3. Results

### 3.1. 6-PPD/6-PPDQ exposure decreases the cell viability of MEFs

**Fig. 1.**
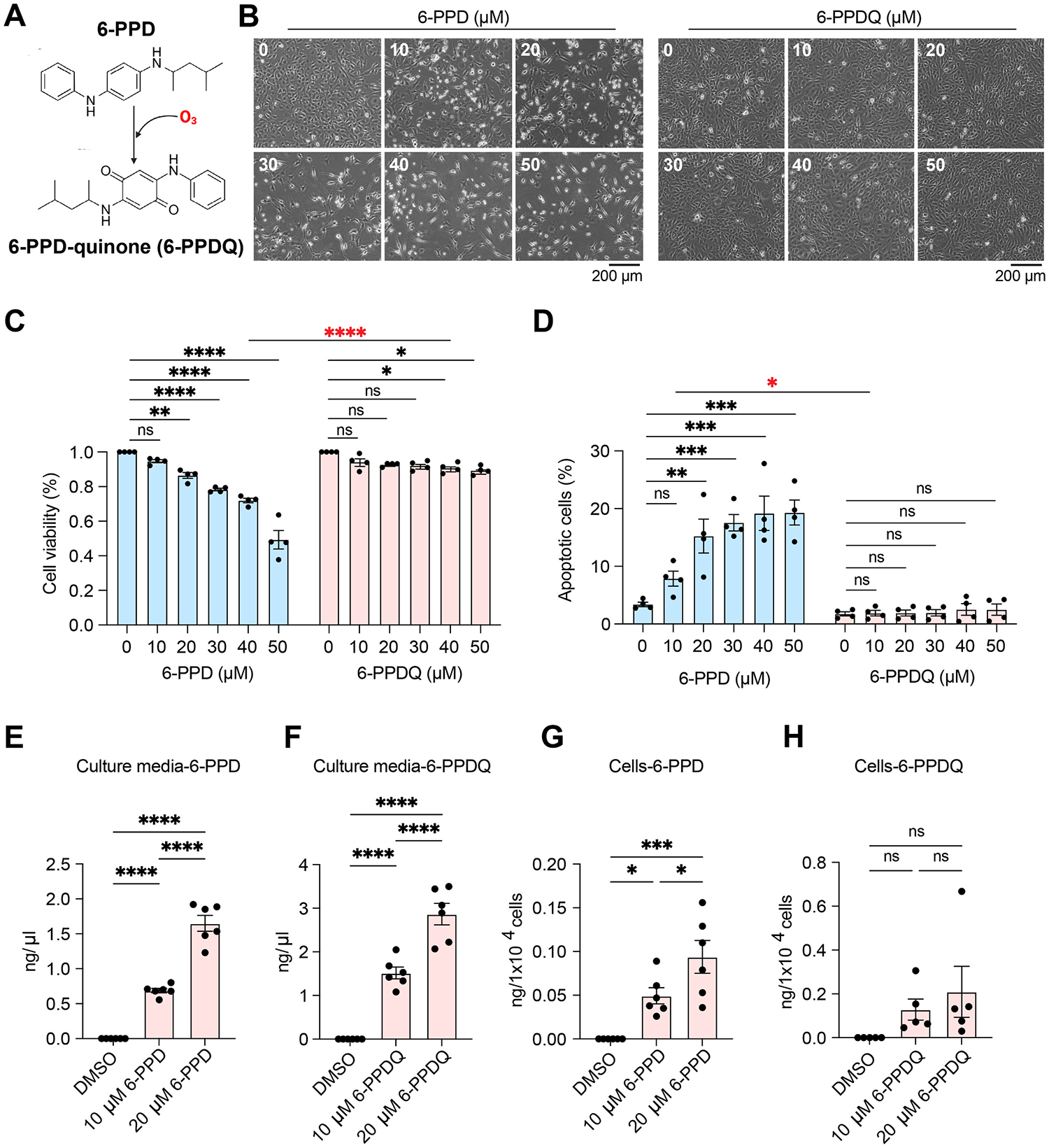
Effects of 6-PPD and 6-PPDQ on MEF Cell Viability and Intracellular Absorption. (A) Transformation of 6-PPD to 6-PPDQ. (B and C) Representative phase-contrast images of MEF cells (B) and MTT assay results (C) following 24-hour exposure to 6-PPD and 6-PPDQ, showing dose-dependent decreases in cell viability. (D) Quantification of apoptotic cells following treatment with 6-PPD or 6-PPDQ. (E-F) HPLC analysis of 6-PPD and 6-PPDQ concentrations in the culture medium following 24-hour exposure to 10 μM or 20 μM of each compound, showing dose-dependent increases. (G-H) Intracellular concentrations of 6-PPD and 6-PPDQ in MEF cells after 24-hour exposure. All data were normalized to corresponding untreated controls. Each dot indicates an individual assay. Data represent the mean ± SEM of at least 3–4 independent assays. *p < 0.05, **p < 0.005, ***p < 0.001, ****p < 0.0001; statistical significance was determined using one-way ANOVA with Tukey’s post hoc test.

MEF cells were exposed to 6-PPD and its derivative 6-PPDQ, which is formed through the oxidation of 6-PPD, at concentrations ranging from 0 to 50 μM for 24 h (Fig. 1A). 6-PPD treatment caused significant cell detachment and morphological changes, including cell rounding and shrinkage, as early as 10 μM (Fig. 1B). In contrast, 6-PPDQ treatment did not induce significant morphological alterations (Fig. 1B). To assess the effects of 6-PPD and 6-PPDQ on cell viability, we performed an MTT assay. The results showed a dose-dependent decrease in cell viability with increasing concentrations of 6-PPD. 6-PPDQ also decreased cell viability when exposed to concentrations higher than 40 μM, but its impact was noticeably weaker than that of 6-PPD at the same concentration (P < 0.0001) (Fig. 1C). Consistent with the MTT assay results, the cell counting kit-8 (CCK-8) assay also revealed a reduction in cell viability upon exposure to both compounds, with 6-PPD exhibiting stronger toxicity (Supplementary Fig. S1). Additionally, a significant increase in apoptotic cells was observed following 6-PPD treatment (Fig. 1D), whereas no such effect was detected with 6-PPDQ exposure. These findings suggest that 6-PPD induces more pronounced cell death effects than 6-PPDQ in MEFs, with increased apoptosis contributing to its toxicity.

### 3.2. Concentration of 6-PPD and 6-PPDQ in MEFs

To investigate whether the milder toxicity of 6-PPDQ compared to 6-PPD is due to reduced cellular absorption, we quantified the concentrations of 6-PPD and 6-PPDQ in both the culture medium and MEF cells after 24-hour exposure to 10 μM or 20 μM of each compound, using HPLC. Analysis of the culture medium 24 h after treatment with 6-PPD did not detect 6-PPDQ. Similarly, only limited levels of 6-PPD were detected in media treated with 6-PPDQ, minimal interconversion between the two compounds in the extracellular liquid environment, consistent with previous studies [105] (Supplementary Fig. S2). Treatment with increasing concentrations of 6-PPD and 6-PPDQ led to a dose-dependent increase in their levels in the culture medium (Figs. 1E, F). In 6-PPD-treated cell lysates, the intracellular concentrations of 6-PPD were 0.049 ± 0.023 ng and 0.094 ± 0.046 ng for 10 μM and 20 μM treatments, respectively (Fig. 1G). In 6-PPDQ-treated cells, the concentrations of 6-PPDQ were 0.13 ± 0.11 ng and 0.21 ± 0.26 ng for 10 μM and 20 μM treatments, respectively (Fig. 1H). The mean absorption rates of 6-PPDQ were 165 % higher at 10 μM and 123 % higher at 20 μM compared to 6-PPD exposure conditions. These findings suggest that the higher toxicity of 6-PPD in MEFs is not primarily due to higher intracellular accumulation compared to 6-PPDQ.

### 3.3. 6-PPD exposure disrupts mitochondrial homeostasis in MEFs

**Fig. 2.**
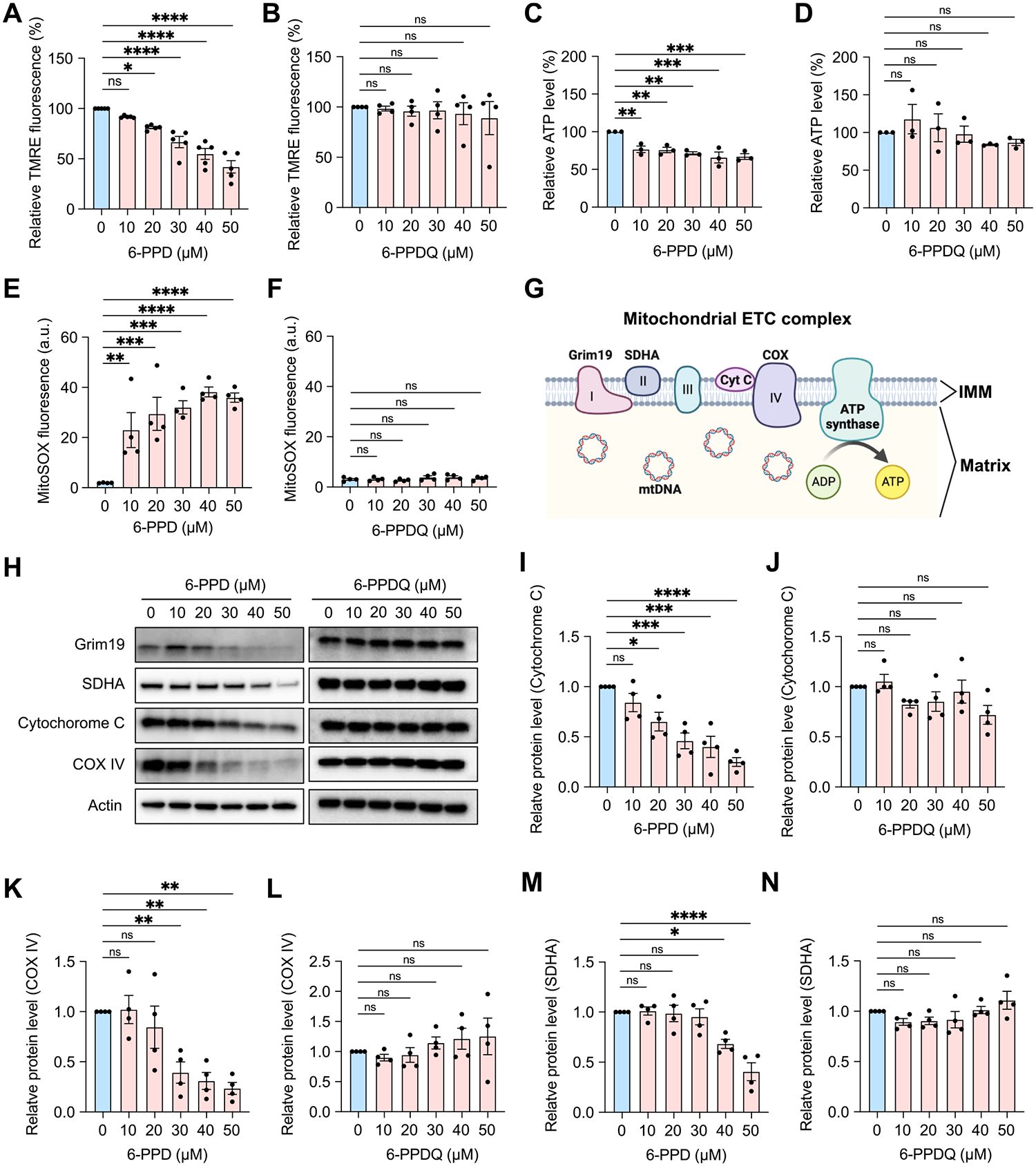
6-PPD exposure disrupts mitochondrial homeostasis in MEFs. (A, B) Quantification of mitochondrial membrane potential (ΔΨm) in MEFs following exposure to increasing concentrations of 6-PPD (A) or 6-PPDQ (B). (C, D) ATP levels in MEFs treated with 6-PPD (C) or 6-PPDQ (D). (E, F) Mitochondrial ROS levels were measured using MitoSOX staining. (G) Diagram illustrating key ETC components in mitochondria. (H) Representative Western blot analysis of ETC proteins in MEFs treated with 6-PPD or 6-PPDQ. (I–N) Quantification of mitochondrial ETC protein levels. Data represent the mean ± SEM. *p < 0.05, **p < 0.005, ***p < 0.001, ****p < 0.0001; statistical significance was determined using one-way ANOVA with Tukey’s post hoc test.

Mitochondria are critical regulators of cell death and are primary targets of various toxicants [73]. To examine the effects of 6-PPD and 6-PPDQ on mitochondrial function in MEF cells, we measured Δψm, ATP production, and mitochondrial reactive oxygen species (ROS). Consistent with survival assay results, 6-PPD exposure caused a dose-dependent reduction in Δψm (Fig. 2A), whereas 6-PPDQ had no significant effect (Fig. 2B). ATP levels were decreased in 6-PPD-treated cells (Fig. 2C) but remained unchanged in 6-PPDQ-treated cells (Fig. 2D). Additionally, mitochondrial ROS levels, assessed using the mitochondrial-specific dye MitoSOX, were significantly elevated in a concentration-dependent manner in 6-PPD-treated cells (Fig. 2E), while no such increase was observed in 6-PPDQ-treated cells (Fig. 2F) [106]. To further explore the impact of 6-PPD on mitochondrial function, we performed a Western blot analysis of key mitochondrial electron transport chain (ETC) proteins (Figs. 2G, H). 6-PPD exposure reduced the protein levels of cytochrome c oxidase (Fig. 2I), COX IV (Fig. 2K), and succinate dehydrogenase subunit A (Fig. 2M). In contrast, 6-PPDQ exposure did not affect these proteins (Figs. 2J, L, and N). Consistent differences in responses to 6-PPD and 6-PPDQ were observed in the levels of Grim-19 in complex I (Supplementary Figs. S3A, B), suggesting that 6-PPD affects the expression of ETC components in MEFs. These findings suggest that 6-PPD significantly disrupts mitochondrial function and induces ROS generation in MEFs.

### 3.4. 6-PPD exposure delays development and reduces reproduction in C. elegans

**Fig. 3.**
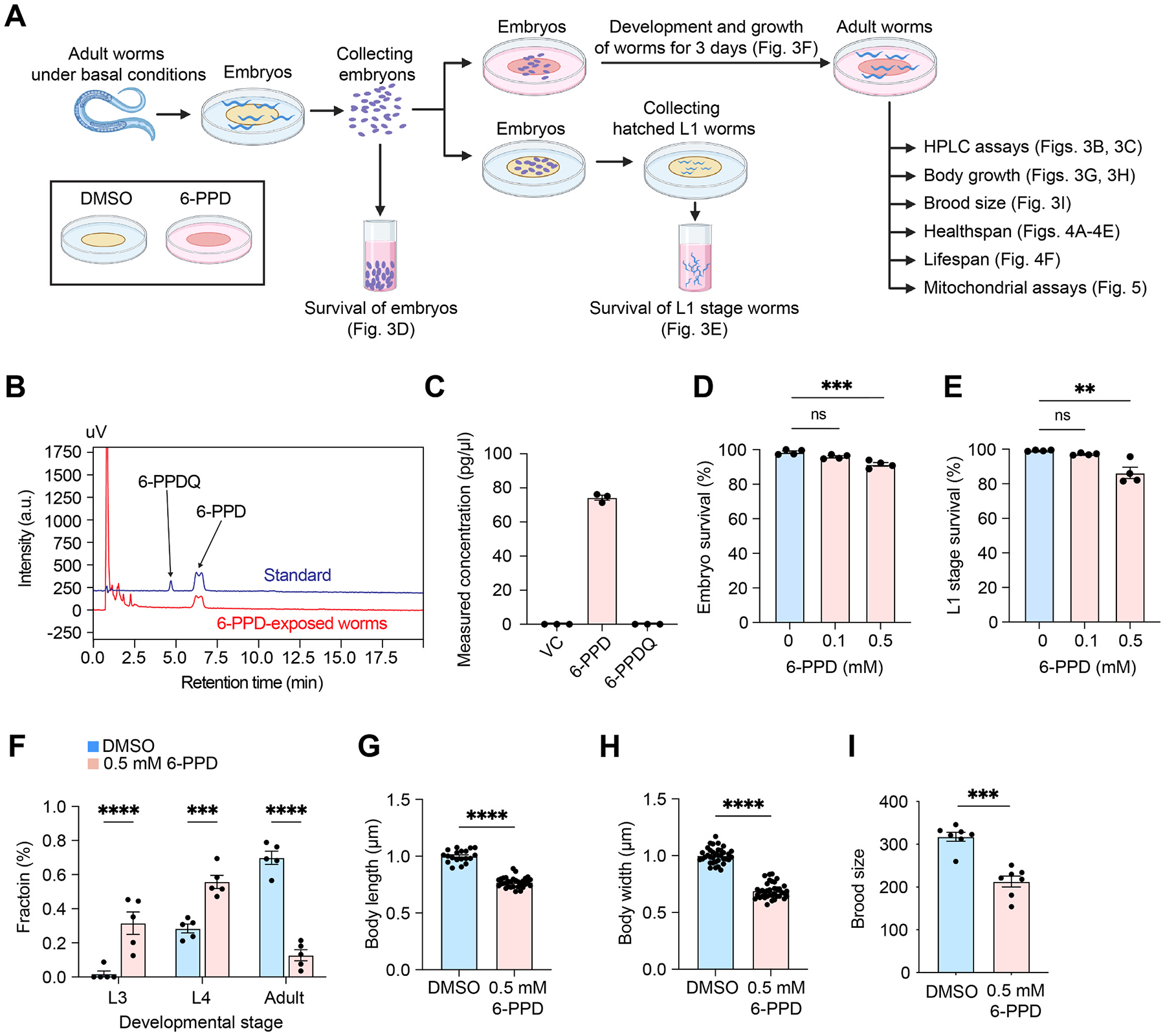
6-PPD exposure impairs development and reproduction in *C. elegans*. (A) Schematic representation of the experimental design. (B) Representative HPLC chromatograms for the detection of 6-PPD and 6-PPDQ in worms exposed to 0.5 mM 6-PPD. (C) Quantification of three independent HPLC analyses. (D and E) Lethality of embryos (D) and L1-stage worms (E) following 6-PPD exposure. (F) Percentage of worms reaching adulthood within 72 h. (G and H) Growth measurements of 6-PPD-treated worms, including body length (G) and body width (H). (I) Brood size analysis showing reduced reproductive capacity in worms exposed to 0.5 mM 6-PPD. Each dot represents an independent group (C-F) or an individual worm (G-I). Data represent the mean ± SEM. *p < 0.05, **p < 0.005, ***p < 0.001, ****p < 0.0001; statistical significance was determined using one-way ANOVA with Tukey’s post hoc test (C-E), two-way ANOVA (F), and two-tailed Mann–Whitney test (G-I).

Intrigued by the toxicity of 6-PPD in cultured cells, we conducted a comprehensive investigation of its physiological effects across the lifespan of live *C. elegans* (Fig. 3A). To measure the uptake and retention level of 6-PPD, we performed HPLC analysis and found that worms exposed to 0.5 mM 6-PPD contained approximately 72.47 pg/μL of 6-PPD, which corresponds to only ~0.055 % of the total applied concentration, indicating limited bioaccumulation under our experimental conditions (Figs. 3B, C, and supplementary Fig. S4). Like the results in MEFs, no detectable level of 6-PPDQ was found, suggesting its concentration is below the limit of detection (5.20 pg/μL) and indicating minimal transformation from 6-PPD to 6-PPDQ within *C. elegans* (Figs. 3B, C). Next, to evaluate the physiological impacts of 6-PPD exposure, we assessed mortality, development, growth, and reproductive capacity (Fig. 3A). Embryos exposed to 0.5 mM 6-PPD exhibited significantly reduced hatching rates compared to controls, and L1-stage worms displayed increased lethality (Figs. 3D, E). As for the long-term effects of 6-PPD exposure on development and growth, 31.5 % of worms treated from the embryonic stage remained at the L3 stage. Only 13 % of these worms reached adulthood within 72 h, compared to 70 % in controls, indicating delayed development (Fig. 3F). Growth measurements confirmed these toxic effects, as 6-PPD-treated worms exhibited significantly reduced body length and width compared to untreated controls (Figs. 3G, H). Additionally, exposed worms produced fewer progeny, suggesting impaired reproductive capacity (Fig. 3I). Together, these findings indicate that 6-PPD exposure disrupts normal development and reproductive fitness in *C. elegans*.

### 3.5. 6-PPD exposure alters the healthspan and lifespan of C. elegans

**Fig. 4.**
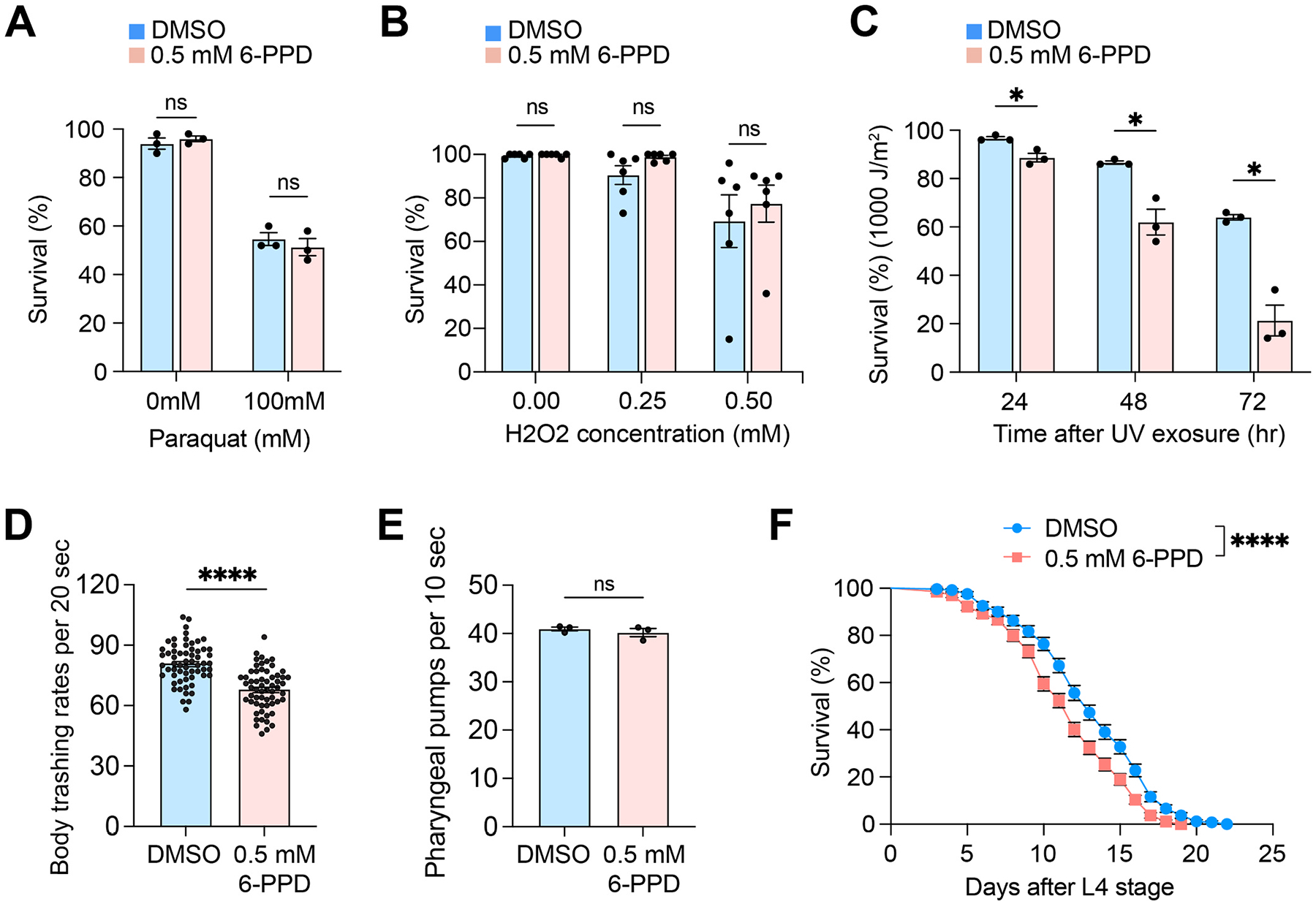
6-PPD exposure alters healthspan and lifespan in *C. elegans*. (A, B) Survival rates under oxidative stress induced by paraquat (A) and hydrogen peroxide (B). (C) Survival rates following UV irradiation stress in worms exposed to 6-PPD. (D) Quantification of body thrashing frequency in liquid medium as a measure of body motility. (E) Pharyngeal pumping rate in 6-PPD-treated and control worms. (F) Lifespan analysis of worms treated with 6-PPD from 1-day-old adulthood. Data are presented as mean ± SEM for all except lifespan, which is shown with SE. Each dot represents an independent group (A, B, C, and E) or an individual worm (D). *p < 0.05, ****p < 0.0001; statistical significance was determined using two-way ANOVA (A-C), Mann–Whitney test (D), Student’s *t*-test (E), and log-rank test for lifespan (F).

We further investigated the impact of 6-PPD exposure on aging by assessing various healthspan parameters. In *C. elegans*, healthspan is defined as the duration of preserved physiological function and stress resistance before age-related decline, typically measured by locomotion, stress response, and tissue integrity [6,9,16,29,30,43,45,54,59,85]. When assessing oxidative stress resistance, we found that exposure to 0.5 mM 6-PPD did not significantly impact survival rates following treatment with paraquat (Fig. 4A) or hydrogen peroxide (Fig. 4B). However, under UV-induced stress, 6-PPD-treated worms were more sensitive, with only 21 % surviving after 72 h compared to 64 % in the control group (Fig. 4C). Worms treated with 0.5 mM 6-PPD exhibited a significant decrease in body thrashing in a liquid medium, which served as an indicator of body motility (Fig. 4D), while pharyngeal pumping rates remained unaffected by 6-PPD exposure (Fig. 4E). Finally, when worms were cultured from the 1-day-old adults to rule out the possibility of lifespan reduction due to developmental defects, the mean lifespan of 6-PPD-exposed worms (11.5 days) was significantly reduced by 31.78 % compared to the control group (7.8 days) (Fig. 4F). Worms exposed to 6-PPDQ had a shorter median lifespan (9 days) than those treated with 6-PPD (10 days, *P* = 0.03), indicating that 6-PPD is less toxic to lifespan than its quinone derivative in *C. elegans*. (Supplementary Fig. 5). Together, these results suggest that 6-PPD negatively impacts both the healthspan and lifespan of *C. elegans*.

### 3.6. 6-PPD exposure impairs mitochondrial function in C. elegans

**Fig. 5.**
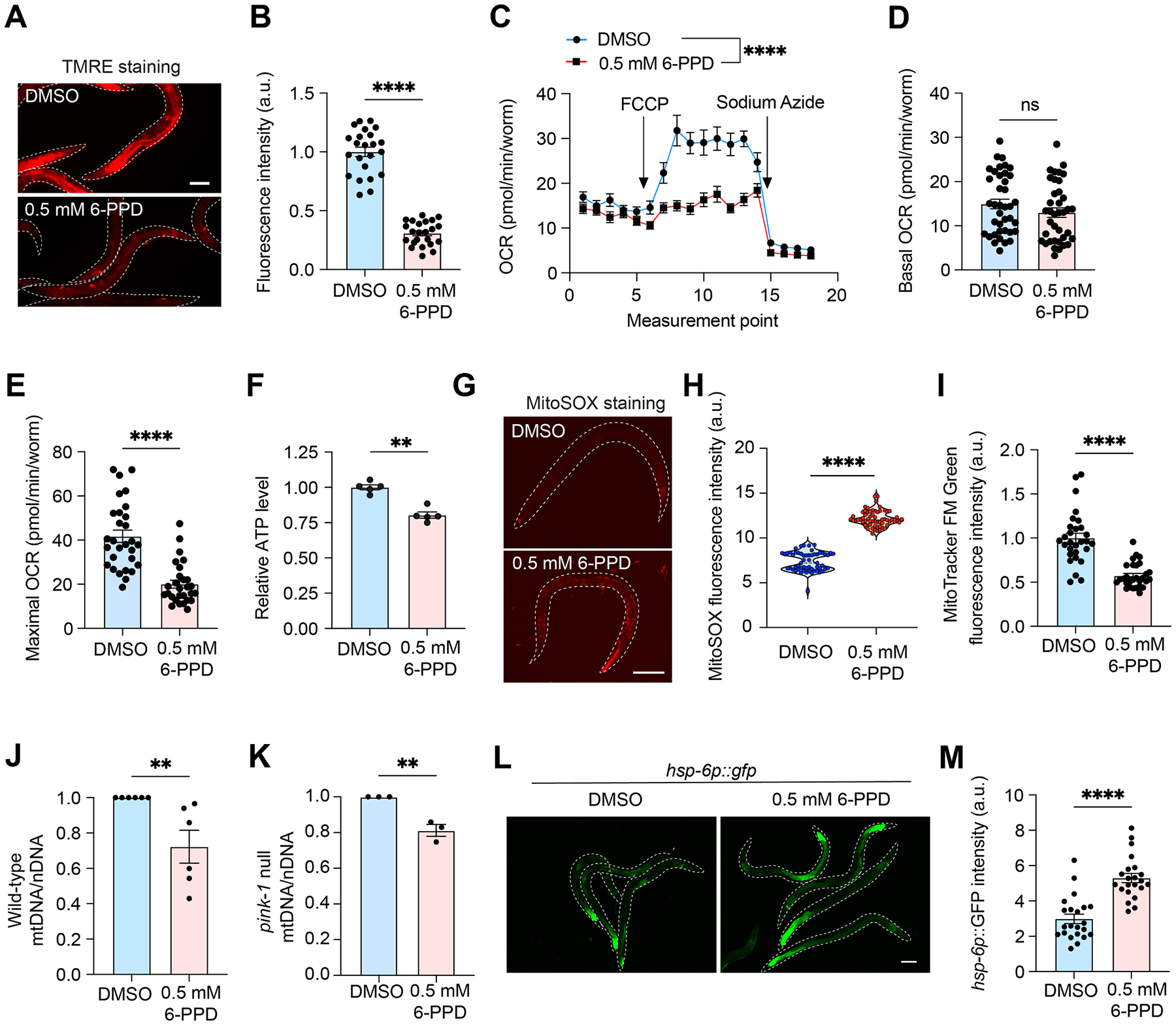
6-PPD exposure disrupts mitochondrial function in *C. elegans*. (A, B) Representative images (A) and quantification (B) of TMRE staining show a significant reduction in mitochondrial membrane potential in 6-PPD-treated worms. (C) OCR measured by Seahorse analysis of live worms with and without 6-PPD exposure. Measurements were taken approximately every 5 min. (D, E) quantification of the basal (D) and maximal OCR (E). Each dot represents a biological replicate of 10 worms from three independent experimental measures. (F) ATP levels are significantly reduced in 6-PPD-treated worms. (G, H) Representative images (G) and quantification (H) of MitoSox intensity indicate increased mitochondrial ROS levels in 6-PPD-treated worms. (I) Relative MitoTracker FM Green fluorescence intensity of the 6-PPD exposed group compared to that in the DMSO control group. (J, K) qPCR analysis of mitochondrial genome copy number in wild-type (J) and *pink-1(tm1779)* null mutant backgrounds (K). (L, M) *hsp-6p::gfp* reporter expression is upregulated in 6-PPD-treated worms, suggesting activation of mitochondrial stress responses. Data represent the mean ± SEM. Each dot represents an independent worm (B, H, I, and M) or an independent group (D, E, F, J, and K). The dashed area indicates the boundary of the worm’s body. Scale bars, 100 μL. Data represent the mean ± SEM. *p < 0.05, **p < 0.005, ***p < 0.001, ****p < 0.0001; statistical significance was determined using Mann–Whitney test (B, D, E, H, I, and M), and Student’s *t*-test (F, J, and K).

Given the detrimental effects of 6-PPD on mitochondrial function in MEFs, we next investigated whether similar disruptions occur in a whole-organism model. To do this, we exposed L1-stage *C. elegans* to 6-PPD until they reached 2-day-old adults and assessed the impact on mitochondrial homeostasis (Fig. 3A). TMRE staining revealed a significant reduction in Δψm following 6-PPD exposure (Figs. 5A, B) [32,79]. Moreover, OCR measurements showed that while basal OCR remained unchanged, maximal OCR was significantly lower in 6-PPD-exposed worms compared to DMSO-treated controls (Figs. 5C–E). Consistently, ATP levels were also reduced (Fig. 5F). Mitochondrial ROS levels, assessed using MitoSOX staining, showed increased fluorescence in the 6-PPD-exposed group, indicating elevated ROS levels (Figs. 5G, H) [106]. Next, we investigated whether the observed disruptions in mitochondrial function were associated with a reduction in mitochondrial quantity. MitoTracker Green FM staining revealed decreased fluorescence in 6-PPD-treated worms, indicating reduced mitochondrial mass (Fig. 5I) [71]. Quantitative polymerase chain reaction (qPCR) analysis also showed a significant decrease in mtDNA copy number in 6-PPD-exposed worms (Fig. 5J). The impaired mitophagy in the *pink-1 (tm1779)* null mutant background failed to prevent this reduction, suggesting that enhanced mitophagy is not likely the primary cause (Fig. 5K). Finally, to assess mitochondrial stress induced by 6-PPD exposure, we measured the expression of the *hsp-6p::gfp* reporter, which is upregulated in response to elevated ROS, mitochondrial dysfunction, and disrupted mitochondrial proteostasis [56,63,86,108]. We found that this reporter was significantly upregulated in 6-PPD-treated worms, further supporting the idea that 6-PPD disrupts mitochondrial homeostasis (Figs. 5L, M). These results demonstrate that 6-PPD exposure impairs mitochondrial homeostasis and increases ROS levels in live worms.

### 3.7. 6-PPD exposure enhances the activity of the conserved SKN-1/Nrf2 transcription factor in C. elegans

**Fig. 6.**
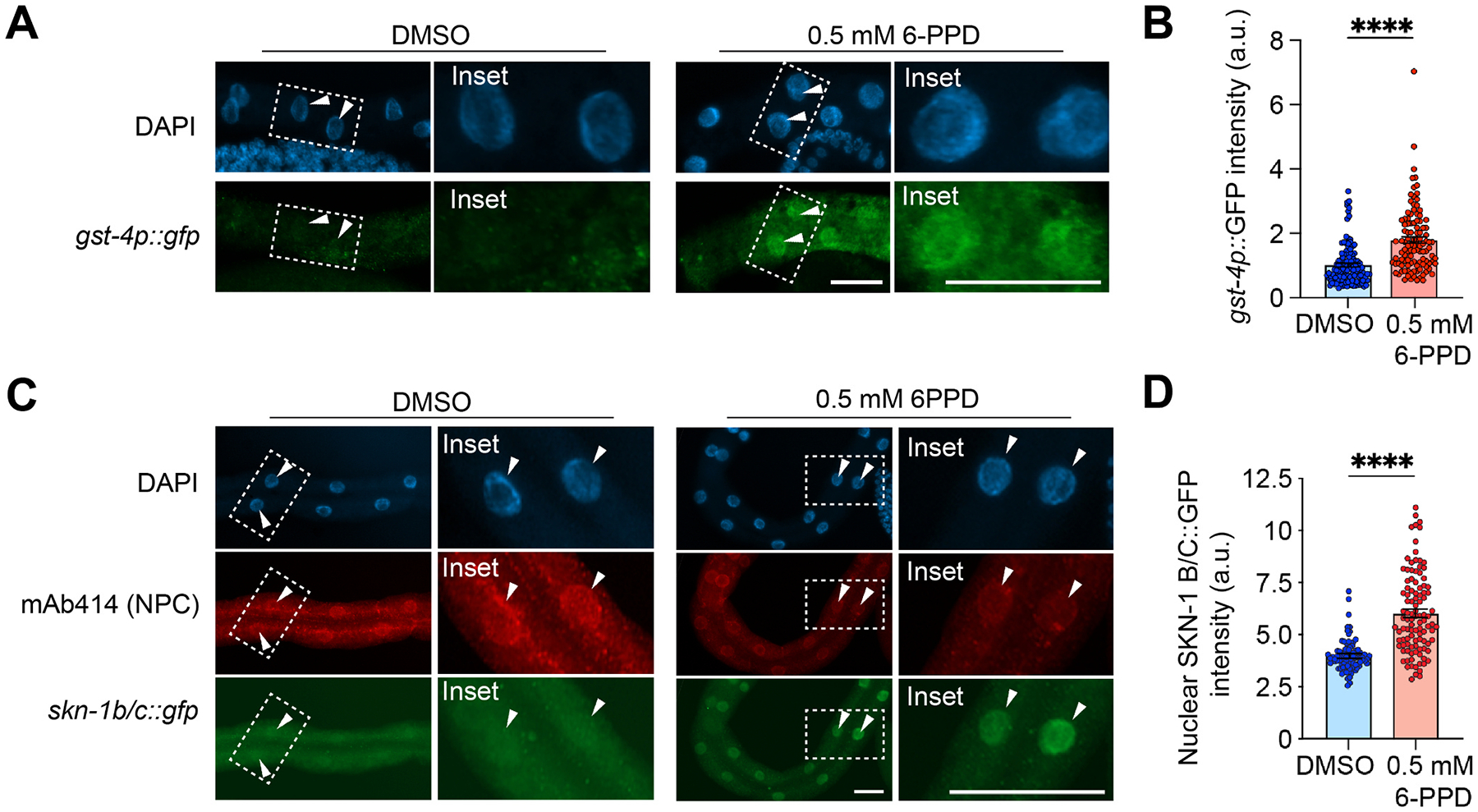
6-PPD exposure increases SKN-1/Nrf2 activation. (A, B) Representative images (A) and quantification (B) of *gst-4p::gfp* reporter fluorescence in the intestine of 6-PPD-treated and control worms. (C, D) Representative images (C) and quantification (D) of SKN-1::GFP nuclear localization in the intestine of 6-PPD-treated and control worms. Scale bars, 20 μL. Data represent the mean ± SEM. Each dot represents an individual worm. ****p < 0.0001; statistical significance was determined using the one-way ANOVA with Tukey’s post hoc test.

We hypothesized that 6-PPD could activate oxidative stress response pathways like SKN-1/Nrf2 [2,4,89,90,94]. SKN-1/Nrf2 is a transcription factor that accumulates in the nucleus in response to oxidative stress, leading to the transcriptional activation of glutathione S-transferase 4 (*gst-4*), which is commonly used as a readout for SKN-1 activity [2,4,49, 53,55]. Using a *gst-4p::gfp* reporter strain to measure SKN-1 activation in the extracted gut, we observed mild but significantly increased fluorescence in the intestine of 6-PPD-treated worms (Figs. 6A, B) [53,55]. *skn-1(zj15)* loss-of-function (lf) mutants failed to induce *gst-4p::gfp* expression, indicating that the response to 6-PPD requires SKN-1 (Supplementary Fig. 6A) [91]. Reverse transcription–qPCR (RT-qPCR) analysis also confirmed a significant SKN-1–dependent upregulation of *gst-4* expression (Supplementary Fig. 6B). Other glutathione S-transferases, such as *gst-5* and *gst-33*, were also elevated following 6-PPD exposure (Supplementary Figs. 6C, D). Furthermore, we observed increased nuclear localization of SKN-1::GFP in the extracted gut (Figs. 6C, D). Collectively, these results suggest that 6-PPD exposure activates the SKN-1/Nrf2 pathway.

### 3.8. SKN-1/Nrf2 activation contributes to 6-PPD-induced toxicity

**Fig. 7.**
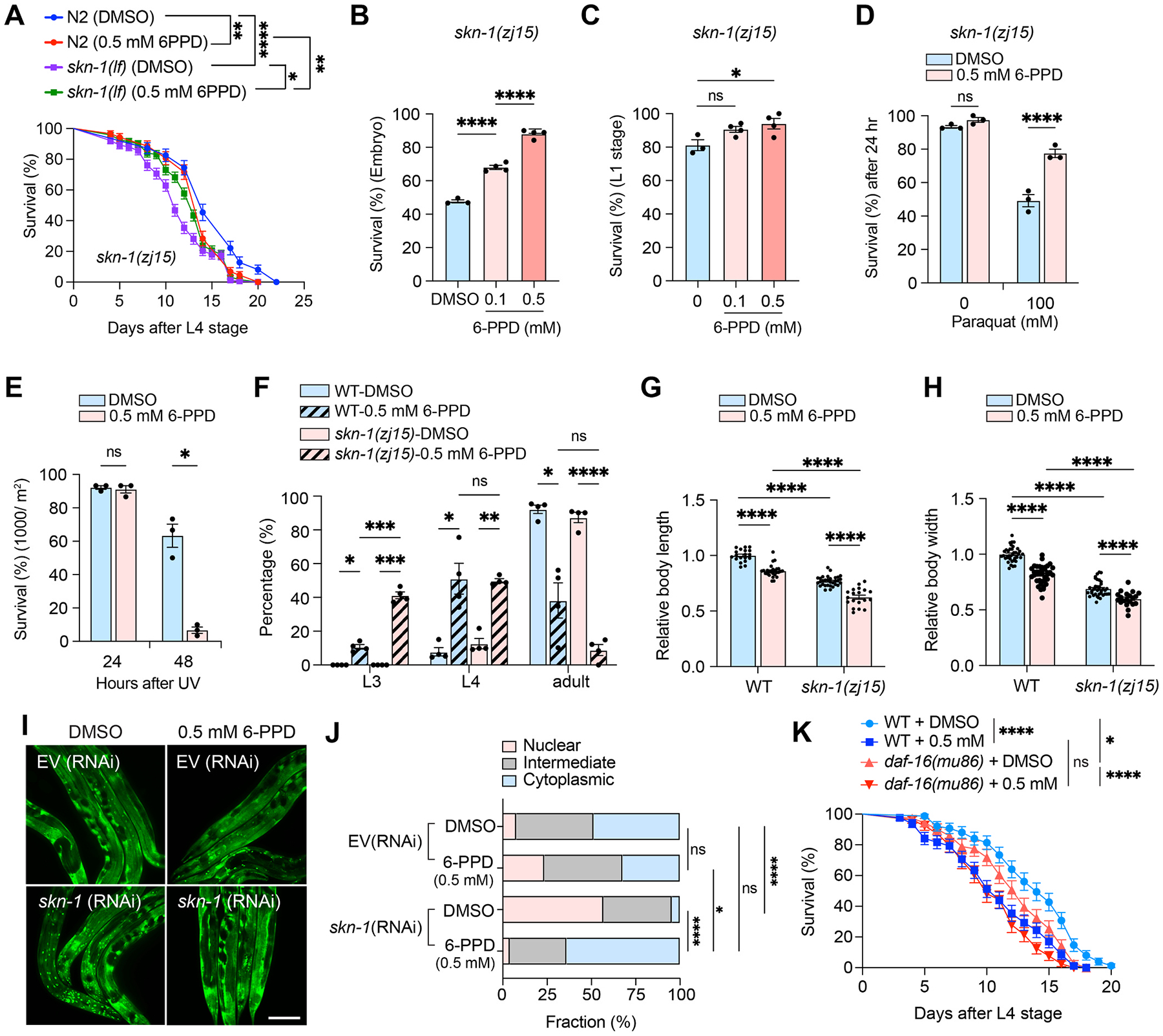
The role of SKN-1/Nrf2 in the physiological response to 6-PPD toxicity. (A) Lifespan analysis of *skn-1(zj15)* mutants treated with 6-PPD or DMSO from day 1 of adulthood. (B) Quantification of egg-hatching rates following 6-PPD exposure in *skn-1(zj15)* mutants. (C) Lethality of L1-stage worms following 6-PPD exposure in *skn-1(zj15)* mutants. (D, E) Quantification of resistance to paraquat-induced oxidative stress (D) and UV irradiation (E) in *skn-1(zj15)* mutants with and without 6-PPD exposure. (F) The fraction of worms reaching adulthood 72 h after egg release, with the number of worms at each developmental stage recorded. (G, H) Quantification of body growth parameters, including body length (G) and width (H). (I) Representative images showing DAF-16/FOXO accumulation in the nuclei. (J) Histogram showing the fraction of worms with cytosolic, intermediate, or nuclear localization of DAF-16::GFP (n = WT + DMSO, 38; WT + 6-PPD, 68; *skn-1*(RNAi) + DMSO, 44; *skn-1*(RNAi) + 6-PPD, 25). (K) Lifespan analysis of *daf-16*(RNAi) mutants treated with DMSO or 0.5 mM 6-PPD. Data are presented as mean ± SEM for all except lifespan, which is shown with SE. Each dot represents an independent group (B-F) or an individual worm (G, H). *p < 0.05, **p < 0.01, ***p < 0.001, ****p < 0.0001; statistical tests: log-rank test for lifespan (A, K), one-way ANOVA (B, C), two-way ANOVA (D-H), and chi-square pairwise (J).

To investigate the physiological role of SKN-1 in mediating 6-PPD-induced alterations in healthspan and lifespan in *C. elegans*, we analyzed the effects of 6-PPD exposure in *skn-1(zj15)* hypomorphic allele [91]. *skn-1(zj15)* mutants have shorter lifespan than wild-type worms [91]. Notably, 0.5 mM 6-PPD exposure increased the lifespan of *skn-1(zj15)* mutants in a dose-dependent manner, in contrast to its lifespan shortening effect in wild-type worms (Fig. 7A and Supplementary Fig. 7A). Interestingly, 6-PPD-induced reduction in hatching rate and increased L1 larval mortality, which were prominent in WT worms, were significantly suppressed in *skn-1(zj15)* mutants (Figs. 7B, C). The *skn-1(zj15)* mutants also exhibited higher resistance to oxidative stress induced by paraquat following 6-PPD exposure compared to the DMSO-treated control condition (Fig. 7D). However, the protective effects of impaired SKN-1/Nrf2 signaling were not observed under UV-induced stress (Fig. 7E). Similarly, developmental rate (Fig. 7F) and body growth (Figs. 7G, H) were unaffected by 6-PPD exposure in *skn-1 (zj15)* mutants. To determine whether upregulation of the antioxidant pathway under SKN-1–activated conditions contributes to SKN-1–mediated effects, we performed RNAi knockdown of *gst-4* and assessed its impact. In contrast to *skn-1* loss-of-function conditions, *gst-4* depletion increased lethality at both the L1 and L4 stages upon 6-PPD exposure, indicating that SKN-1–dependent activation *of gst-4* plays a protective, rather than detrimental, role against 6-PPD–induced toxicity (Supplementary Figs. 7B–D). Collectively, these findings suggest that SKN-1/Nrf2 activation is associated with the toxic effects of 6-PPD on survival, lifespan, and oxidative stress tolerance, while its involvement in developmental delay and stress resistance is selective.

### 3.9. 6-PPD negatively regulates DAF-16/FOXO

Recent studies indicate that SKN-1 acts as a negative regulator of somatic stress resistance and modulates the activity of the DAF-16/FOXO transcription factor [20]. DAF-16 functions through nuclear translocation in response to oxidative stress, activating the expression of target genes, including superoxide dismutase, such as *sod-3* [20,22,75,92]. We found that 6-PPD exposure did not significantly alter *sod-3* expression (Supplemental Fig. 8A) [20,22,75,92]. Similarly, 6-PPD did not significantly increase DAF-16 nuclear localization in adult worms (Figs. 7I, J and Supplementary Fig. 8B). Consistent with prior reports, *skn-1* RNAi significantly enhanced DAF-16 nuclear localization (Figs. 7I, J). Notably, 6-PPD treatment suppressed this increase (Figs. 7I, J). The reduced lifespan observed in both 6-PPD–exposed wild-type worms and *daf-16(mu86)* null mutants was not further shortened under combined conditions (Fig. 7K). Furthermore, the absence of DAF-16 did not further exacerbate the 6-PPD–induced reduction in developmental delay (Supplemental Fig. 8C) or brood size (Supplementary Fig. 8D). Together, our data suggest that DAF-16 activity is not induced by 6-PPD and may be either suppressed or unresponsive under these conditions, contributing to its limited role in protecting worms against 6-PPD toxicity.

### 3.10. SKN-1 depletion does not suppress the 6-PPD-induced mitochondrial dysfunction

**Fig. 8.**
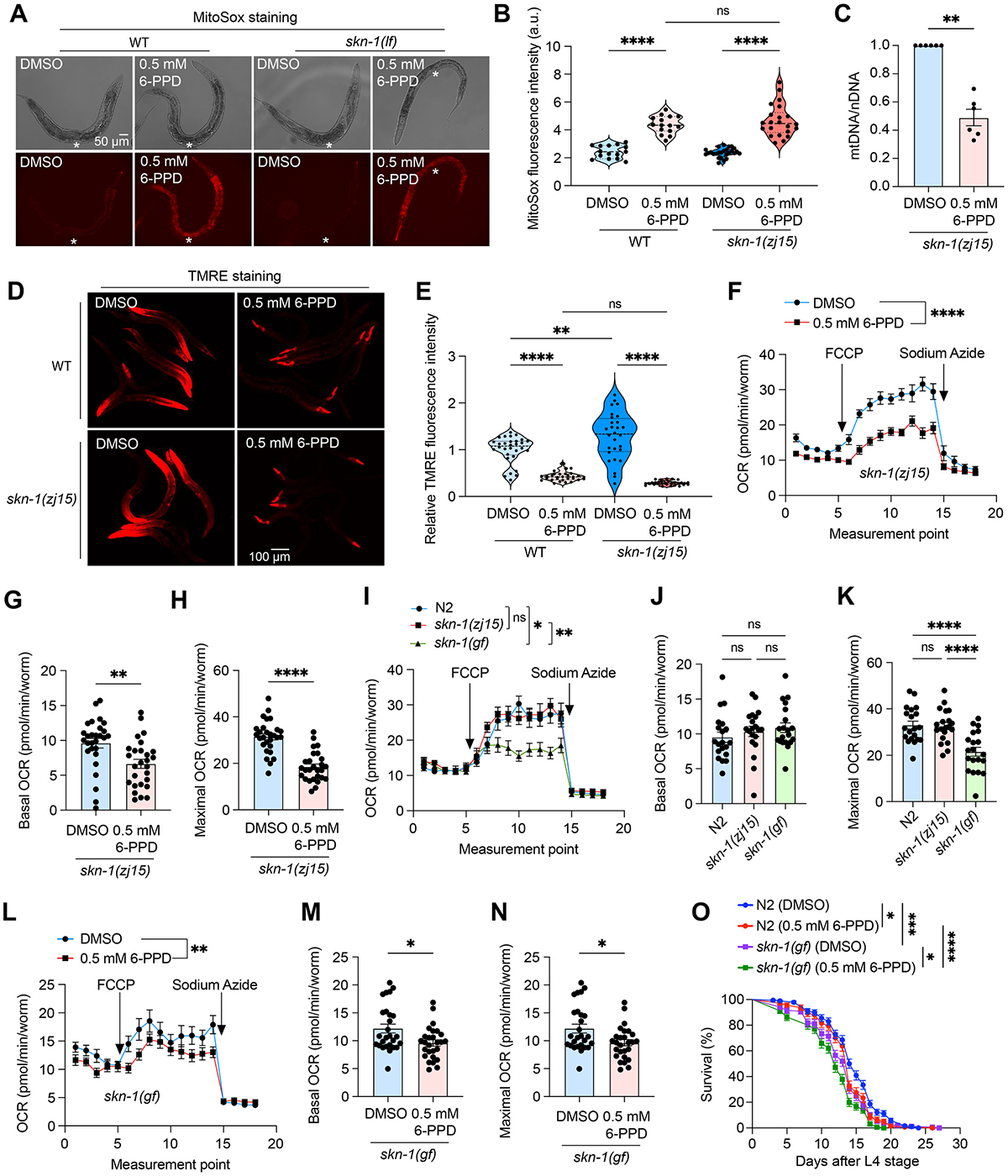
Impacts of SKN-1/Nrf2 depletion on 6-PPD-induced mitochondrial dysfunction. (A, B) Representative images (A) and quantification (B) of MitoSox staining showing mitochondrial superoxide levels. Asterisks in (A) indicate the vulva. Each dot in (B) represents a single worm. (C) qPCR analysis of mitochondria genome copy number. (D, E) Representative images (D) and quantification (E) of TMRE staining showing mitochondrial membrane potential levels. Each dot in (E) represents a single worm. (F) OCR measured by Seahorse analysis in *skn-1(zj15)* lf mutants with and without 6-PPD exposure. Measurements were taken approximately every 5 min. (G, H) Basal (G) and Maximal (H) OCR in *skn-1(zj15)* lf mutants with and without 6-PPD exposure. Each dot represents a biological replicate of approximately 10 worms from three independent measures. (I) OCR in *skn-1(zj15)* lf and *skn-1(lax188)* gf mutants with only DMSO exposure. Measurements were taken approximately every 5 min. (J, K) Basal (J) and maximal (K) OCR in (I). Each dot represents a biological replicate of approximately 10 worms from two independent experimental measures. (L) OCR in *skn-1(lax188)* gf mutants with and without 6-PPD exposure. Measurements were taken approximately every 5 min. (M, N) Basal (M) and maximal (N) OCR in *skn-1(zj15)* gf mutants with and without 6-PPD exposure. Each dot represents a biological replicate of approximately 10 worms from three independent measures. (O) Lifespan analysis of *skn-1(lax188) gf* mutants treated with 0.5 mM 6-PPD or DMSO. *p < 0.05, **p < 0.01, ***p < 0.001, ****p < 0.0001; statistical tests: two-way ANOVA (B, E), Mann–Whitney (F-H and L-N), Student’s *t*-test (C), one-way ANOVA (I-K), and log-rank test for lifespan (O). Data are presented as mean ± SEM for all except lifespan, which is shown with SE.

To further investigate the relationship between 6-PPD exposure and SKN-1 activity, we examined whether enhanced SKN-1 function contributes to the mitochondrial defects observed in 6-PPD-treated animals. In contrast to the suppression of lifespan shortening by 6-PPD exposure observed in *skn-1(zj15)* mutants, elevated mitochondrial ROS levels following 6-PPD exposure were not significantly reduced in *skn-1(zj15)* mutants compared to WT animals (Figs. 8A, B). Similarly, the decreased mtDNA content (Fig. 8C) and Δψm levels were not restored in *skn-1 (zj15)* mutants (Figs. 8D, E). In addition, *skn-1(zj15)* mutants still exhibited significantly reduced OCR after 6-PPD exposure (Figs. 8F–H). These results suggest that 6-PPD impairs mitochondrial function either exclusively through an SKN-1-independent pathway or through a combination of both SKN-1-dependent and SKN-1-independent pathways.

To further test this possibility, we assessed mitochondrial function in *skn-1(lax188)* gain-of-function (gf) mutants [77]. Even under basal conditions (DMSO treatment only), *skn-1(gf)* mutants exhibited reduced mitochondrial OCR compared to both wild-type and *skn-1(lf)* animals (Figs. 8I–K). Consistent with these findings, an independent set of measurements further confirmed that *skn-1(gf)* mutants exhibited reduced basal and maximal OCR compared to WT controls, under untreated conditions (Supplementary Fig. 9). These results indicate that constitutive SKN-1 activation is sufficient to impair mitochondrial function. Furthermore, treatment of *skn-1(gf)* mutants with 6-PPD led to an additional decline in mitochondrial function and a further reduction in lifespan (Figs. 8L–N), supporting the conclusion that 6-PPD impairs mitochondrial health through mechanisms that are at least partially SKN-1-independent. In line with these observations, 6-PPD-treated *skn-1 (gf)* mutants exhibited an exacerbated reduction in lifespan compared to untreated controls (Fig. 8O).

Collectively, these findings suggest that 6-PPD impairs mitochondrial function and reduces organismal lifespan through both SKN-1-dependent and SKN-1-independent mechanisms. Moreover, while SKN-1 activation contributes to mitochondrial dysfunction, it is not the sole driver of 6-PPD-induced mitochondrial damage.

## 4. Discussion

The mortality of adult coho salmon following exposure to 6-PPDQ in stormwater has raised significant concerns regarding the environmental toxicity of 6-PPD and 6-PPDQ in aquatic species [93]. Recent studies have expanded our understanding of the toxic effects of 6-PPDQ on non-aquatic species, including *C. elegans* and mammals [48,96]. 6-PPD toxicity in aquatic species has also been documented [25,26]. However, its impact on non-aquatic species, particularly across the lifespan of animals, remains underexplored.

Our findings using MEF cell cultures and *C. elegans* suggest that 6-PPD is highly toxic. While 6-PPDQ has only a mild effect on cell viability even at high concentrations and does not induce apoptosis or noticeable mitochondrial defects in MEFs, 6-PPD causes significant mitochondrial damage, cell death, and apoptosis (Figs. 1B–D). Consistent with our findings, a study on the rotifer *Brachionus calyciflorus* showed that 6-PPD had a higher toxic effect on rotifers than 6-PPDQ [57]. However, research on zebrafish larvae indicated that 6-PPDQ is more lethal at a lower concentration than 6-PPD, although both compounds exhibited weaker toxic effects compared to their impact on coho salmon [95]. Markedly, they induced distinctive developmental defects and corresponding genomic mRNA expression linked to their respective effects [109]. A recent study further showed that 6-PPD, but not 6-PPDQ, causes severe eye damage in zebrafish by disrupting the thyroid signaling pathway, again supporting their distinct toxic profiles [13].

Our HPLC analysis in MEFs indicates that intracellular 6-PPD concentrations are lower than those of 6-PPDQ, suggesting that higher absorption of 6-PPD is not the cause of its greater toxicity than 6-PPDQ. Furthermore, our analyses indicate minimal conversion of 6-PPD to 6-PPDQ within MEFs and liquid medium. Similarly, in *C. elegans*, 6-PPDQ was not detected, with levels below the detection limit (5.20 pg/μL) (Figs. 3B, C), further supporting limited *in vivo* conversion. These results indicate that the observed toxicity of 6-PPD is independent of its transformation to 6-PPDQ. Our results are supported by several studies that investigated the transformation of 6-PPD to 6-PPDQ under different environmental conditions. In the aquatic environment, the transformation of 6-PPD is minimal, while 6-PPDQ undergoes rapid photodegradation, reducing its toxicity. Additionally, 6-PPDQ predominantly accumulates in TWPs in an ambient atmosphere, with a 1.28 % mass yield from 6-PPD dissipation [105]. Similarly, another study estimated the conversion of 6-PPD to 6-PPDQ with a 9.7 % molar yield for pure 6-PPD and a 0.95 % yield for 6-PPD in TWPs, suggesting these values represent the minimum conversion under the given conditions and time scales [35]. Moreover, the conversion rate of 6-PPD to 6-PPDQ in the body is estimated to be less than 2 % [96]. This suggests that exposure to 6-PPD may pose potential health risks due to the toxicity of 6-PPD itself rather than the conversion of 6-PPD into 6-PPDQ within an organism. It also suggests that different cell types and species have varying susceptibilities to 6-PPD and 6-PPDQ exposures. However, we cannot rule out the possibility that other transformation products from 6-PPD ozonation contribute to these phenotypes. Recent studies show that 6-PPD generates not only 6-PPDQ but also 19 other products, suggesting that its physiological effects may be more complex than those caused by 6-PPDQ alone [35].

We observed that 6-PPD impairs mitochondrial function in MEFs by lowering Δψm (Fig. 2A) and ATP levels (Fig. 2C), increasing ROS production (Fig. 2E), and reducing the expression of ETC components (Figs. 2H–N). Similarly, in *C. elegans*, 6-PPD exposure decreased Δψm (Figs. 5A–B), OCR (Figs. 5C–E), ATP levels (Fig. 5F), and mtDNA copy number (Fig. 5J), while increasing ROS levels (Figs. 5G–H) and upregulating mitoUPR (Figs. 5L–M). Mitochondria are crucial for energy production, redox balance, and stress adaptation and are primary targets of various toxicants [27,38,43]. Their dysfunction can lead to broad physiological effects, including impaired development, healthspan, and lifespan [14,74]. A recent study by Hua and Wang highlighted 6-PPDQ’s mitochondrial toxicity in *C. elegans*, showing that exposure at environmentally relevant concentrations reduces key ETC components, decreasing lifespan [43]. MitoUPR activation occurs at 0.1 and 1 μg/L of 6-PPDQ but is inhibited at 10 μg/L [43]. Our study on the mitochondrial toxicity of 6-PPD in *C. elegans* found that exposure to 0.5 mM 6-PPD triggers upregulation of *hsp-6p::gfp* expression, which is commonly used as a mitoUPR reporter [56,63,86,108] (Fig. 5L). This result is consistent with previous findings from 6-PPDQ, suggesting that both compounds may share similar mitochondrial toxicity [43]. However, the contribution of altered mitoUPR to 6-PPD toxicity remains to be determined, and additional studies are required to elucidate its mechanistic relevance.

While our results show no evidence supporting the toxic effects of 6-PPDQ on mitochondrial homeostasis in MEFs (Fig. 2), other studies in zebrafish and *Daphnia magna* reveal that both 6-PPD and 6-PPDQ triggered mitochondrial dysfunction, though the responses varied by species and exposure duration [1,25,95]. Interestingly, both 6-PPD and 6-PPDQ were observed to increase rather than decrease oxygen consumption rates in zebrafish larvae in a dose-dependent manner [95]. Additionally, exposure to 6-PPDQ (1 and 10 μg/L) in *C. elegans* increases oxygen consumption rate but decreases ATP content, whereas, in our study, 6-PPD exposure decreased both oxygen consumption and ATP content [38]. These results suggest that mitochondrial responses to these compounds may vary across species and environmental conditions. Nevertheless, the mitochondrial alterations observed in both species suggest that mitochondria are a key target organelle of 6-PPD and 6-PPDQ.

An interesting finding in our study was the role of the SKN-1/Nrf2 pathway in mediating 6-PPD toxicity. SKN-1/Nrf2 is well known for promoting detoxification and antioxidant defense, and under stress, it accumulates in the intestinal nucleus to drive these processes [2,4,89, 90,94]. It also plays a key role in longevity regulation and has been implicated in various human diseases [2]. Our findings suggest that chronic exposure to 6-PPD leads to maladaptive activation of SKN-1/Nrf2, as evidenced by upregulation of the *gst-4p::gfp* reporter (Figs. 6A, B, and supplementary Fig. 6B) and increased nuclear translocation of SKN-1 (Figs. 6C, D). Interestingly, *skn-1(zj15)* hypomorphic mutants showed increased resistance to 6-PPD-induced lifespan shortening (Fig. 7A), increased lethality (Figs. 7B, C), and oxidative stress sensitivity (Fig. 7D), indicating that persistent activation of SKN-1/Nrf2 contributes to defects in growth and reproduction. While SKN-1/Nrf2 is crucial for acute oxidative stress resistance, its chronic and unregulated activation in *C. elegans* appears to have complex effects on overall health and aging, including reduced longevity [69,76,77,81,90]. Moreover, *C. elegans* carrying *skn-1(gf)* alleles exhibit age-dependent depletion of fat, reduced body length, and decreased brood size [69,90]. The chronic activation of SKN-1 leads to premature loss of muscle function and reduced longevity [77,81]. Further evidence supports this, as inhibition of WDR-23, a key repressor of SKN-1, significantly reduces resistance to various stresses, including heat, endoplasmic reticulum stress, and mitochondrial stress [20,68]. In mammals, constitutive activation of NRF2 in cancer cells promotes proliferation and chemoresistance through phase II detoxification pathways and pro-survival gene expression, suggesting its balanced regulation is critical for maintaining cellular and organismal health [104]. Notably, *skn-1* (gf) conditions in *C. elegans* are also associated with the diminished activity of the DAF-16/FOXO transcription factor, which plays a central role in regulating stress responses and longevity [20]. Our findings showed no activation of DAF-16/FOXO upon 6-PPD exposure. This may, in part, result from increased SKN-1 activation by 6-PPD, which suppresses DAF-16/FOXO function, suggesting a potential antagonistic interaction between these two pathways under 6-PPD exposure. Moreover, the observation that 6-PPD suppresses DAF-16/FOXO activation even in *skn-1* (lf) mutants indicates that 6-PPD may inhibit DAF-16/FOXO through both SKN-1–dependent and SKN-1–independent mechanisms (Figs. 7I, J). Consistent with this, the lack of additive or synergistic effects of 6-PPD exposure and DAF-16 depletion on brood size, developmental timing, and lifespan further supports the idea that 6-PPD and DAF-16 act within a common pathway (Fig. 7K and Supplementary Figs. 8C, D). However, the mechanisms underlying the enhanced tolerance to 6-PPD in *skn-1* mutants remain unclear and warrant further investigation. Interestingly, previous studies in *C. elegans* have revealed that 6-PPDQ exposure activates DAF-16/FOXO, providing another line of evidence for the distinct impacts of 6-PPD and 6-PPDQ in *C. elegans* [38,43].

Our results suggest that 6-PPD impairs mitochondrial function through both SKN-1-dependent and SKN-1-independent mechanisms. Given that chronic SKN-1 activation is sufficient to interfere with mitochondrial respiration (Figs. 8I–K), SKN-1 activation could be the upstream cause of mitochondrial impairment. However, the lack of mitochondrial rescue including reductions in Δψm (Figs. 8D, E), mtDNA content (Fig. 8C), and OCR (Figs. 8F–H) in *skn-1(zj15)* hypomorphic mutants suggests that activation of *skn-1* alone is not sufficient to account for the mitochondrial damage caused by 6-PPD and additional pathways also contribute to the impairment and its incomplete rescue. One possibility is that 6-PPD or its reactive metabolites may directly damage mitochondrial components, such as respiratory chain complexes, mitochondrial DNA, or membrane lipids, thereby impairing bioenergetic function independently of SKN-1. Although the direct impact of 6-PPD on mitochondria remains unclear, recent studies have shown that its transformation product, 6-PPDQ, disrupts mitochondrial homeostasis in *C. elegans* through multiple mechanisms, even at environmentally relevant concentrations. It inhibits Complex I and II activities, downregulates ETC components, and reduces ATP production while increasing oxygen consumption and ROS levels [38]. Additionally, 6-PPDQ perturbs leucine metabolism by limiting its uptake and accelerating its breakdown, thereby reducing the availability of NADH and FADH_2_ for the respiratory chain [100]. The observed activation of mitoUPR following 6-PPD exposure suggests it may contribute to the compound’s mitochondrial toxicity (Figs. 5L–M). While mitoUPR activation initially promotes mitochondrial protection by inducing chaperones and proteases, chronic overactivation can impair mitochondrial biogenesis and suppress ETC complex expression, leading to decreased oxidative phosphorylation and ATP production, worsening mitochondrial dysfunction [72]. Consistent with this, our results show reduced ETC protein expression in MEFs (Fig. 2 and supplementary Fig. 3) and impaired mitochondrial biogenesis in *C. elegans* (Fig. 5). Therefore, sustained mitoUPR induction in 6-PPD-treated worms likely reflects maladaptive stress, contributing to persistent mitochondrial impairment and reduced organismal health as a SKN-1-independent pathway.

Finally, the concentrations of 6-PPD and 6-PPDQ used in cultured cells and *C. elegans* in this study were markedly higher than those typically found in environmental samples or detected in the human body [11,19,21,24,33,34,50,51,61,83,87,88,99,111]. It has been noted that much higher external concentrations than environmentally relevant levels are required in *C. elegans* to elicit observable toxic effects. This is likely due to the worm’s robust xenobiotic defense systems, including a tough cuticle and highly efficient efflux mechanisms that limit chemical absorption and actively expel xenobiotics [10,18,46,62,82]. Consistently, our HPLC analysis of *C. elegans* showed that despite treating the worms with relatively high concentrations of 6-PPD (0.5 mM), the amounts detected in *C. elegans* were 74.27 pg/μL (Fig. 3B), which is approximately 0.055 % of the exposure dose. Nevertheless, the internal 6-PPD level in worms remains substantially higher than levels detected in human samples, such as 0.068 pg/μL in pregnant women’s urine and 0.057 pg/μL in serum from healthy individuals [24,88]. Previous studies by Dr. Dayong Wang’s group have demonstrated that *C. elegans* exposed to environmentally relevant concentrations of 6-PPDQ (e.g., 10 μg/L) exhibit shortened lifespans, with median values ranging from approximately 9–12 days [41,43]. Consistently, our lifespan assays comparing 0.5 mM 6-PPD and 6-PPDQ revealed that 6-PPDQ reduced the median lifespan to 9 days, which falls within the range reported by Wang’s group, even though they used much lower, environmentally relevant doses (Supplementary Fig. S5). One plausible explanation is that 6-PPDQ toxicity reaches a saturation threshold, beyond which higher external concentrations do not result in proportionally increased toxicity. This also highlights the robust xenobiotic defense mechanisms in *C. elegans*, which both restrict chemical uptake and elicit strong stress responses to counteract the diverse environmental toxicants present in the soil [7,84]. However, we acknowledge that the use of such high concentrations represents a limitation of our study. Future investigations should aim to better characterize 6-PPD accumulation across different environmental and biological contexts and evaluate how environmentally relevant or human-relevant concentrations influence *C. elegans* physiology and stress responses.

In conclusion, our study highlights the significant toxicity of 6-PPD in both MEFs and *C. elegans*, which differs from the toxicity of 6-PPDQ observed in MEFs. We provide evidence that 6-PPD disrupts mitochondrial function and induces SKN-1/Nrf2 activation, leading to impairments in growth, reproduction, and lifespan in *C. elegans*. These findings demonstrate the consistent toxic effects of 6-PPD across two models and emphasize the critical role of the SKN-1/Nrf2 pathway in mediating these effects. Additionally, our results stress the importance of considering species-specific responses when assessing the toxicological impact of environmental contaminants. Further studies are required to determine the role of the Nrf2 signaling pathway in mediating 6-PPD toxicity in mammals.

### Environmental implications

The widespread use of 6-PPD in rubber products and its transformation into 6-PPDQ raise major environmental and public health concerns. Detected in air, water, soil, and human tissues, these compounds show broad bioavailability. Our study reveals that 6-PPD causes mitochondrial dysfunction, oxidative stress, and reduced lifespan in *C. elegans* and mammalian cells. Mechanistically, 6-PPD activates the conserved SKN-1/Nrf2 pathway, contributing to toxicity. Mitochondrial impairment persists even without SKN-1, indicating both SKN-1-dependent and -independent mechanisms. These findings underscore the need for regulatory attention and safer alternatives to mitigate ecological and human health risks.

## Supplementary Material

1

2

## Data Availability

Data will be made available on request.

## HIGHLIGHTS

- 6-PPD causes dose-dependent cell death, apoptosis, and mitochondrial dysfunction in mouse embryonic fibroblasts (MEFs).
- *C. elegans* exposed to 6-PPD exhibit impaired development, reduced reproduction, and shortened lifespan.
- 6-PPD exposure increases the SKN-1/Nrf2 pathway, contributing to early mortality and reduced lifespan in *C. elegans*.
- 6-PPD induces mitochondrial dysfunction through both SKN-1/Nrf2-dependent and -independent mechanisms in *C. elegans*.

## Appendix A. Supporting information

Supplementary data associated with this article can be found in the online version at doi:10.1016/j.jhazmat.2025.140332.
