# Supplementary material for: 6-PPD induces mitochondrial dysfunction and reduces healthspan and lifespan through SKN-1 in Caenorhabditis elegans
Source: J Hazard Mater. Author manuscript; Available in PMC 2026 Jun 18. (PMC13277346; doi:10.1016/j.jhazmat.2025.140332)
Supplement: 1 [file NIHMS2185873-supplement-1.docx]

**
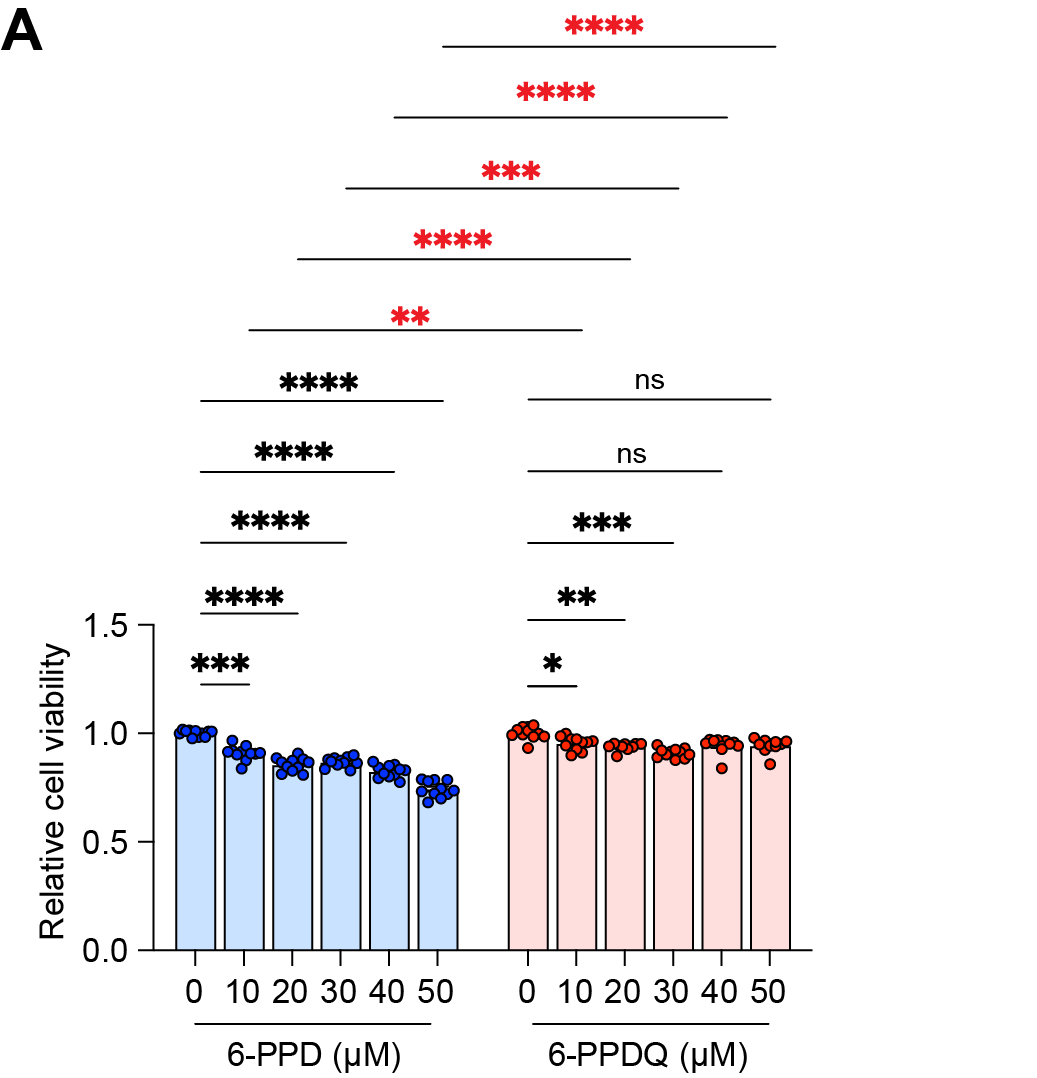
**

**Figure S1. Evaluation of cell viability in MEFs treated with 6‑PPD or 6‑PPDQ.**

MEFs were exposed to increasing concentrations of 6‑PPD or its transformation product 6‑PPDQ (0–50 μM) for 24 h, and cell viability was assessed using the Cell Counting Kit‑8 (CCK‑8) assay. Data are presented as relative viability normalized to untreated controls (mean ± SEM, n = 3 independent experiments). Statistical significance was determined by two‑way ANOVA (*p < 0.05, **p < 0.01, ***p < 0.001, ****p < 0.0001; ns, not significant).


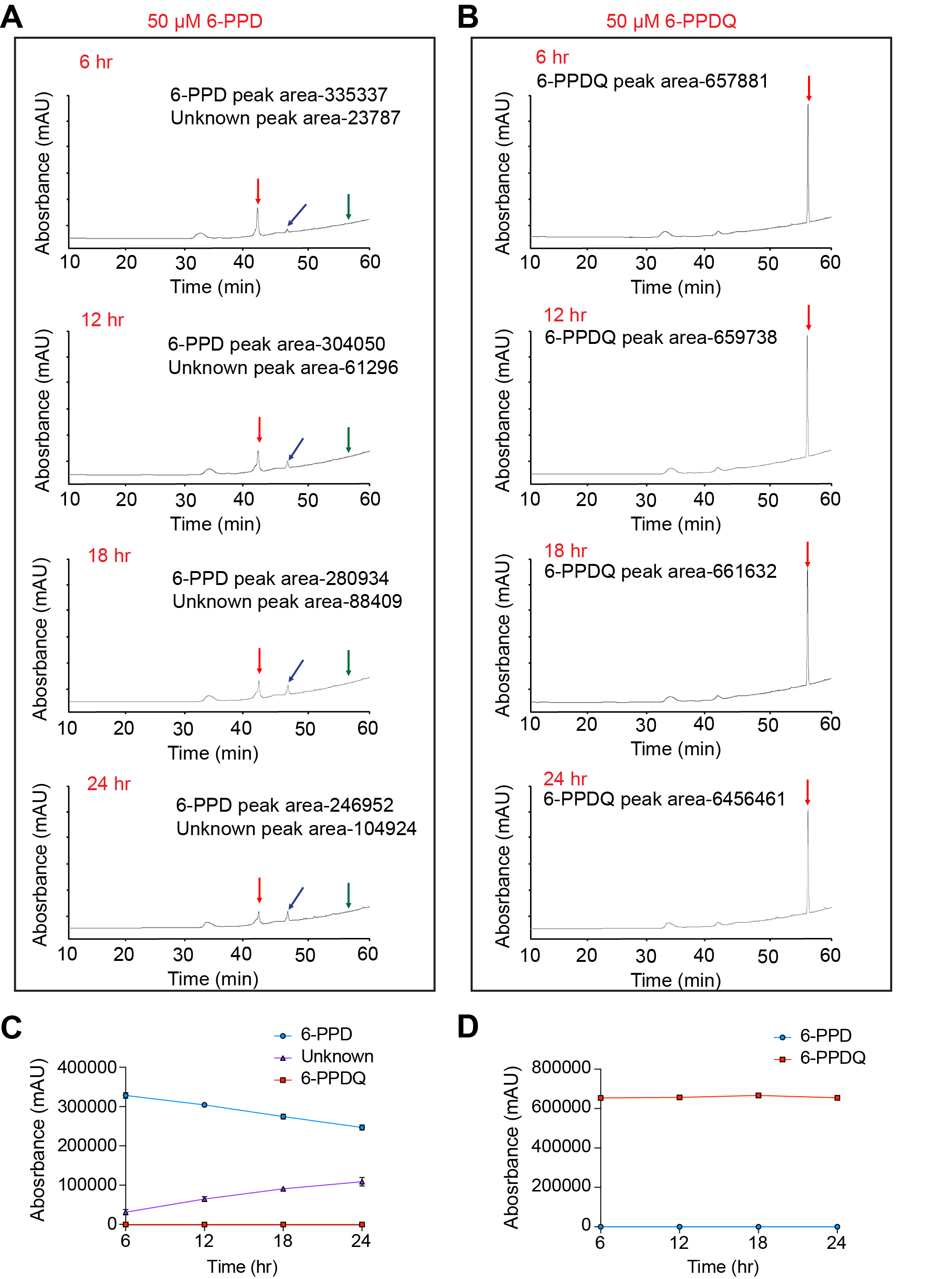


**Figure S2. The transformation between 6-PPD and 6-PPDQ in a liquid media environment.** Standard HPLC analysis confirmed the retention times of 6-PPD at 41.398 min and 6-PPDQ at 56.117 min. (A) HPLC chromatogram of 50 µM 6-PPD in liquid culture media without cells. Red arrows indicate the 6-PPD peak, while green arrows mark the 6-PPDQ peak, which was minimally detected. Purple arrows denote unknown molecular peaks that increased over time. (B) HPLC chromatogram of 6-PPDQ in liquid media following 6-PPD treatment. Red arrows indicate 6-PPDQ peaks, while purple arrows represent the 6-PPD peak, which remained stable during incubation. (C) Quantification of detected 6-PPD levels in the 6-PPD-treated medium. 6-PPD levels decreased over time, accompanied by an increase in an unknown molecule. 6-PPDQ levels were below the limit of detection. (D) Quantification of detected 6-PPD and 6-PPDQ levels in the 6-PPDQ-treated medium. 6-PPD levels were below the limit of detection.

**
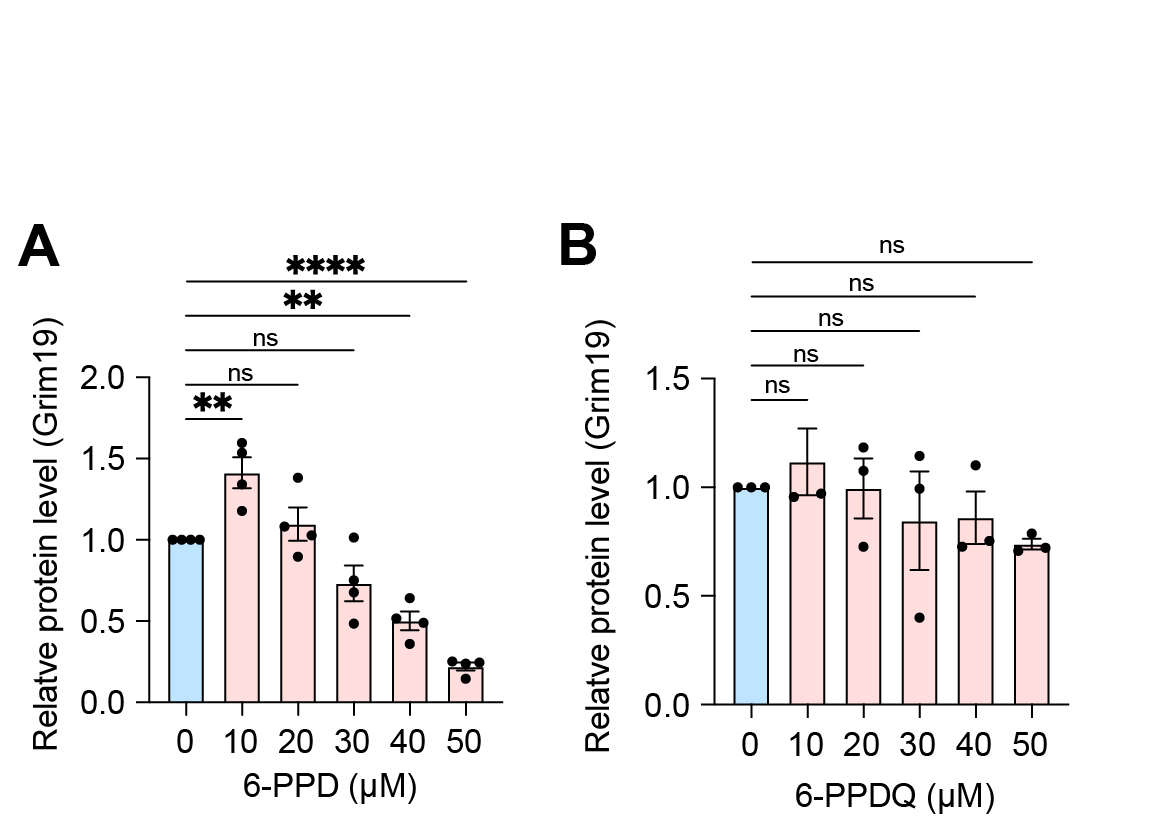
**

**Figure S3. Quantification of mitochondrial ETC protein levels in MEFs in MEFs treated with 6-PPD or 6-PPDQ.** (A, B) Grim 19, an ETC complex I component in 6-PPD (A) and 6-PPDQ (B) exposed MEFs. *p < 0.05, **p < 0.005, ***p < 0.001, ****p < 0.0001; Statistical significance was determined using the one-way ANOVA with Tukey’s post hoc test.


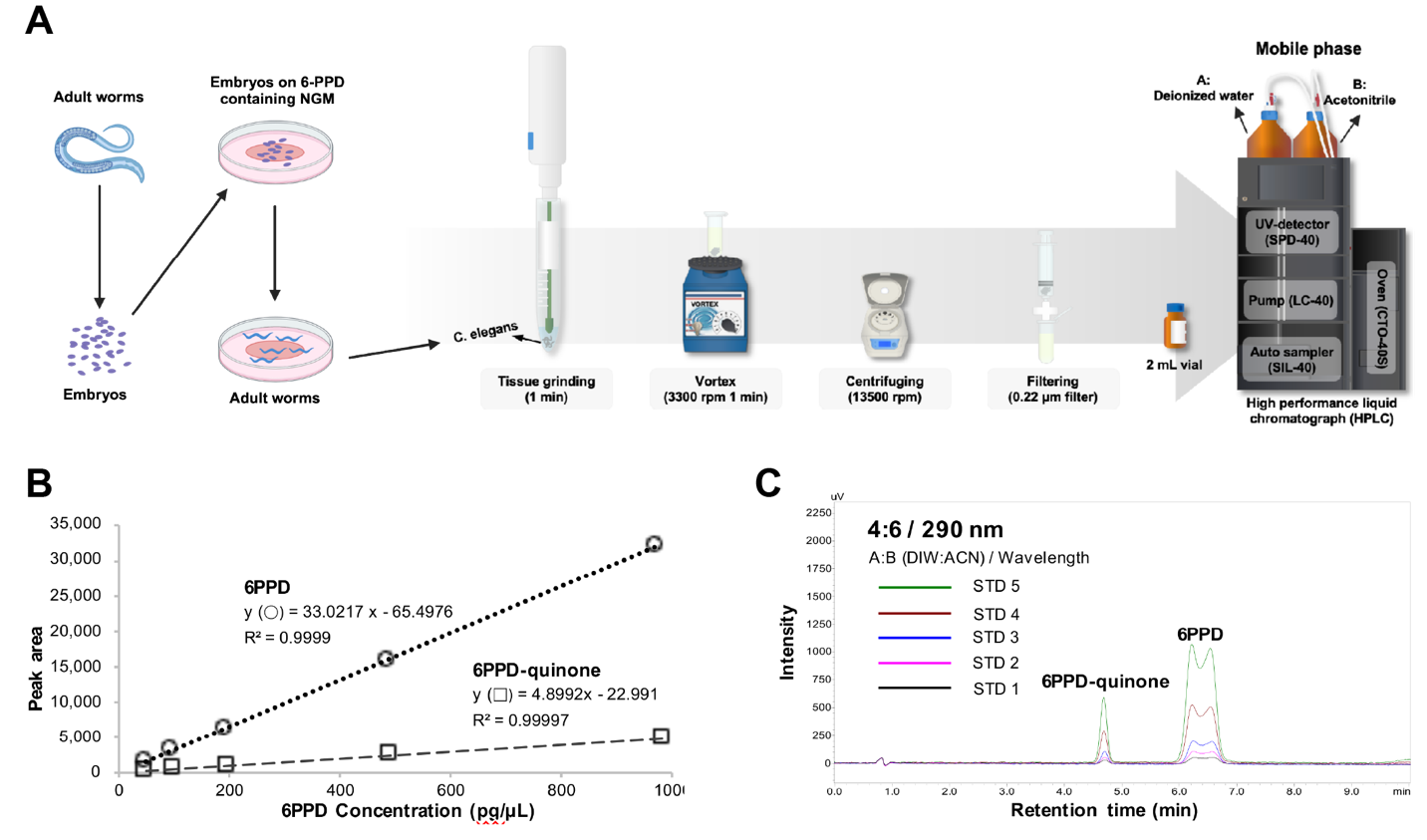


**Figure S4. Scheme for the quantification of 6-PPD and 6-PPDQ in *C. elegans*.**

(A) Pretreatment process of *C. elegans* exposed to 6-PPD and 6-PPDQ. (B) Five-point calibration curves of 6-PPD and 6-PPDQ within the sample-matched calibration range. (C) Chromatograms of standard solutions used for calibration.


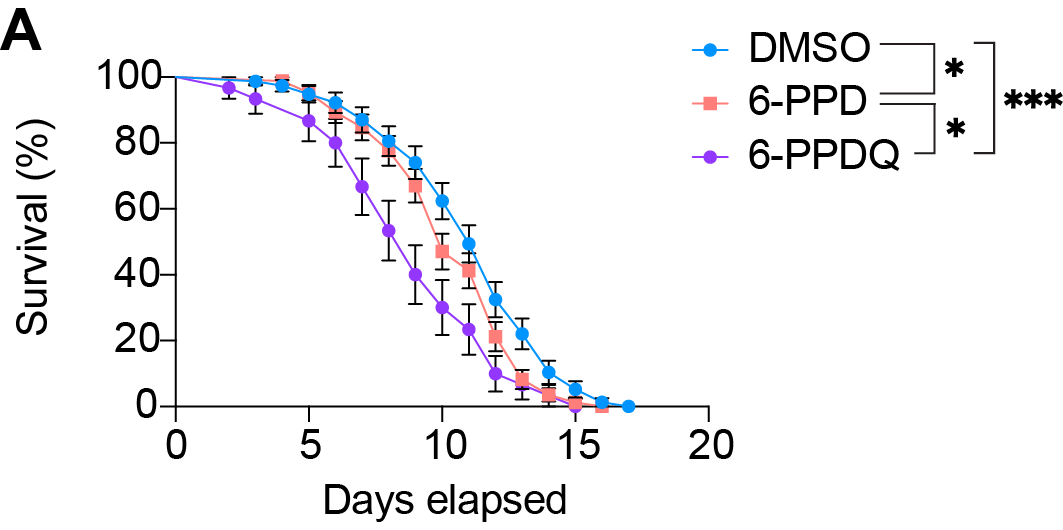


**Figure S5. 6‑PPD and 6‑PPDQ exposures reduce lifespan in *C. elegans*.**

(A) Survival rates of worms treated with DMSO, 6‑PPD, or 6‑PPDQ from the L4 stage to adulthood. Data are presented as mean ± SEM. Statistical significance was determined using the log‑rank (Mantel–Cox) test. *p < 0.05, ***p < 0.001**.**

**
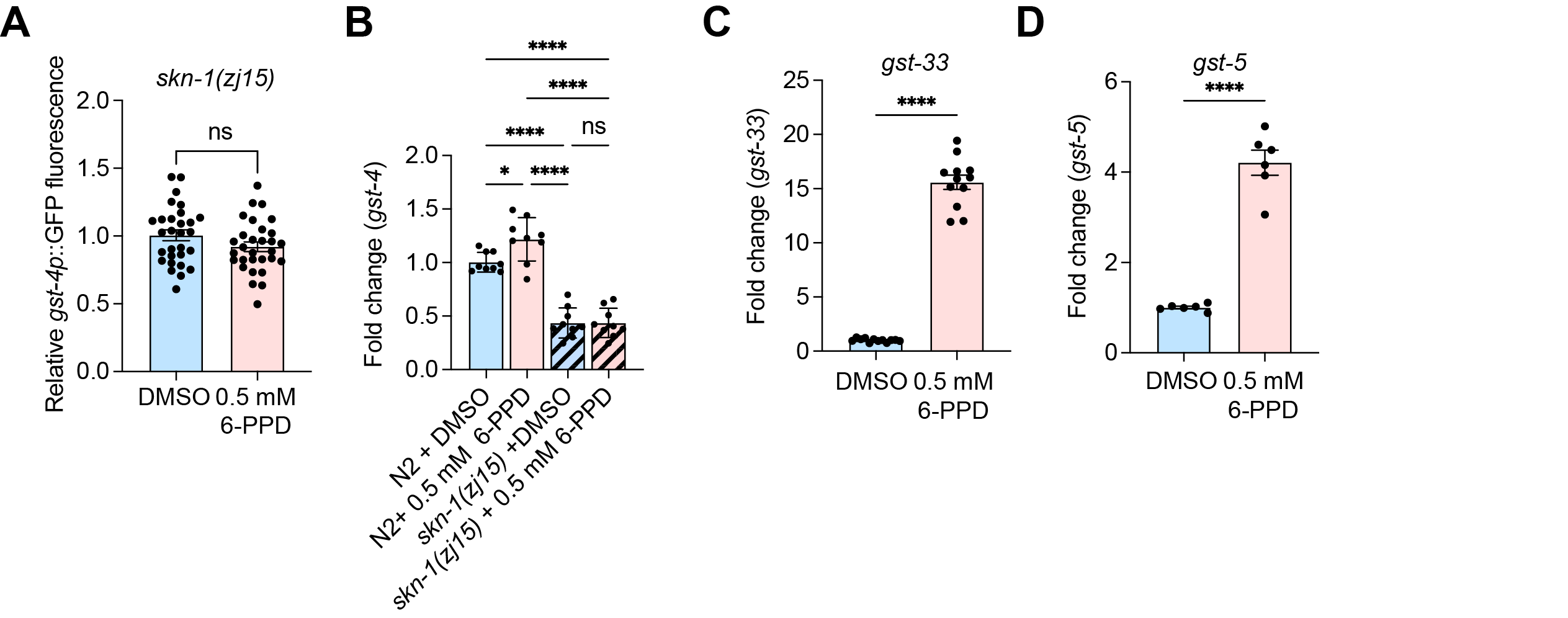
**

**Figure S6. 6‑PPD exposure increases gst‑4 expression in a SKN‑1-dependent manner and affects other antioxidant genes in *C. elegans*.**

(A) Representative images of *gst-4p::GFP* expression in *skn-1(zj15)* hypomorphic mutants treated with or without 0.5 mM 6‑PPD. Each dot represents an individual worm. (B) RT‑qPCR analysis of *gst-4* expression in wild-type and *skn-1(zj15)* worms with or without 0.5 mM 6‑PPD. Each dot represents an individual group. (C, D) RT‑qPCR analysis of *gst-33* (C) and *gst-5* (D) under the same conditions. Data are presented as mean ± SEM. Statistical significance was determined using the Mann–Whitney test (A, C, D) or one-way ANOVA (B). *p < 0.05, ***p < 0.001, ****p < 0.0001.

**
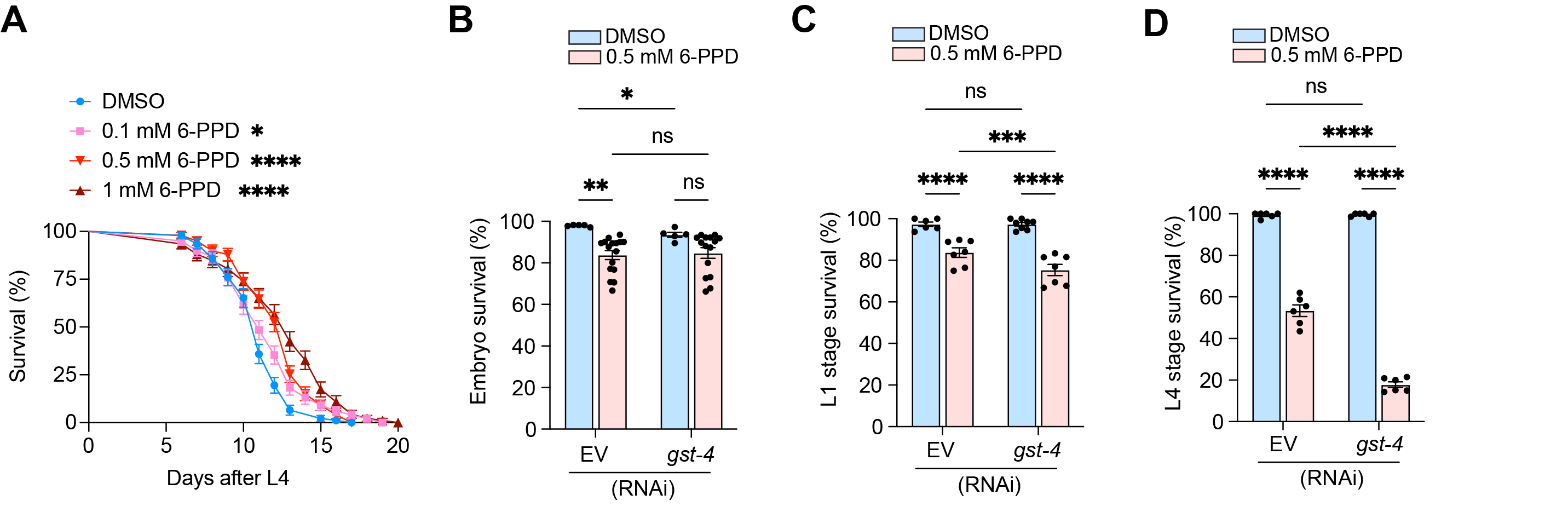
**

**Figure 7. Loss of GST‑4 reduces tolerance to 6‑PPD in *C. elegans*.**

(A) Lifespan analysis of *skn-1(zj15)* hypermorphic mutants treated with 6-PPD from 1-day-old adulthood. (B-D) Lethality of embryos (B), L1-stage worms (C), and L4-stage worms (C) following 6-PPD exposure. *p < 0.05, ****p < 0.0001; statistical significance was determined using two-way ANOVA (A-C), Mann–Whitney test (D), Student’s t-test (E), and log-rank test for lifespan (F). *p < 0.05, **p < 0.005, ***p < 0.001, ****p < 0.0001; Statistical significance was determined using the log‑rank (Mantel–Cox) test (A), and two-way ANOVA (B-D)

**
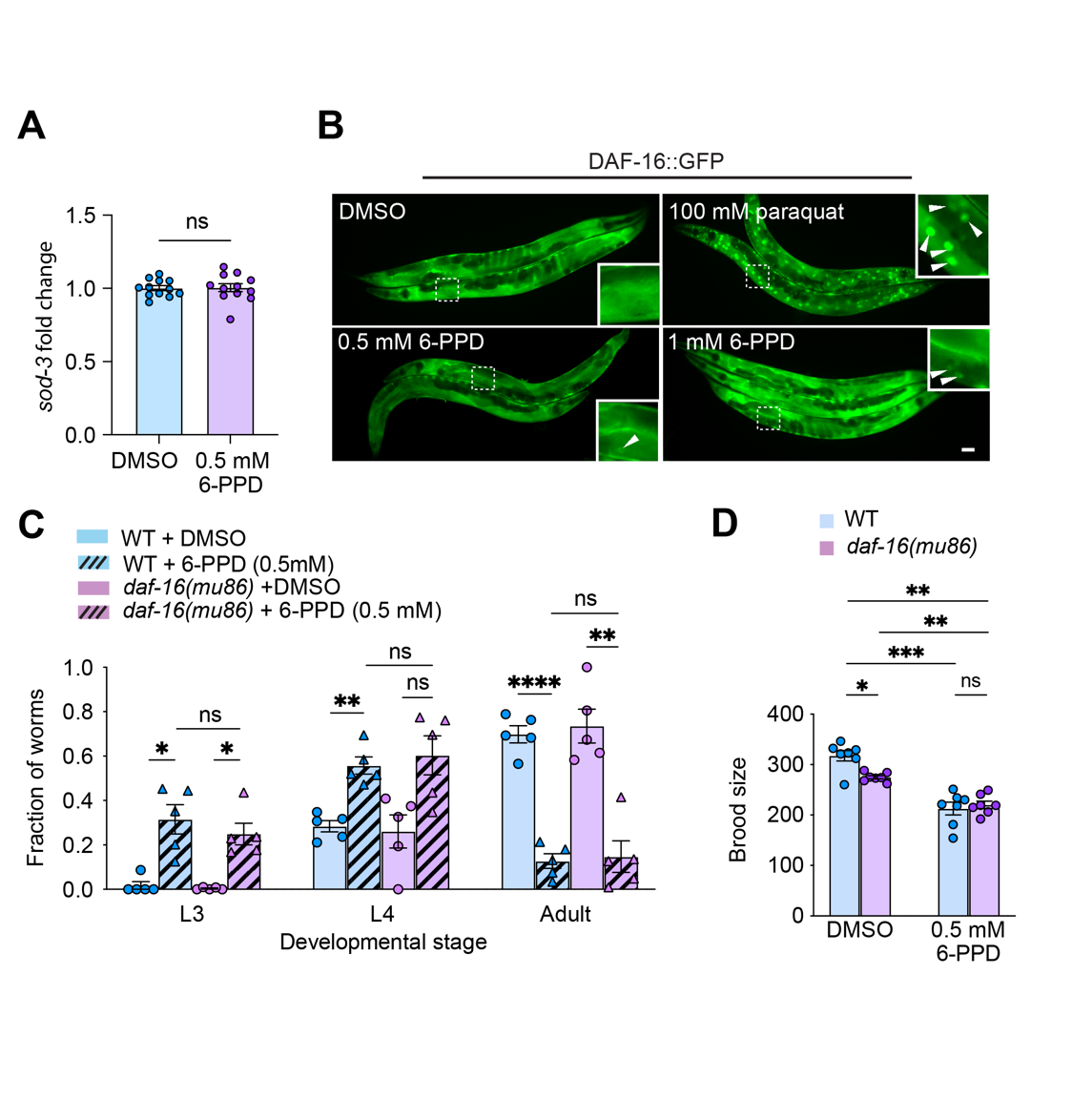
**

**Figure S8. The role of DAF-16/FOXO in worms exposed to 6-PPD.**

(A) qPCR quantification of sod-3, a downstream target of DAF-16/FOXO, following 6-PPD exposure. (B) Representative images showing DAF-16/FOXO accumulation in intestinal nuclei. While paraquat increases nuclear accumulation of DAF-16/FOXO, 6-PPD exposure does not appear to induce a similar effect. (D) The *daf-16* null mutation did not noticeably affect developmental retardation induced by 6-PPD exposure. (C) The *daf-16* null mutation did not alter the reduction in brood size caused by 6-PPD exposure. Each dot represents the value of an individual group. *p < 0.05, **p < 0.005, ***p < 0.001, ****p < 0.0001; Statistical significance was determined using the Mann–Whitney test (A), two-way ANOVA (C, D).


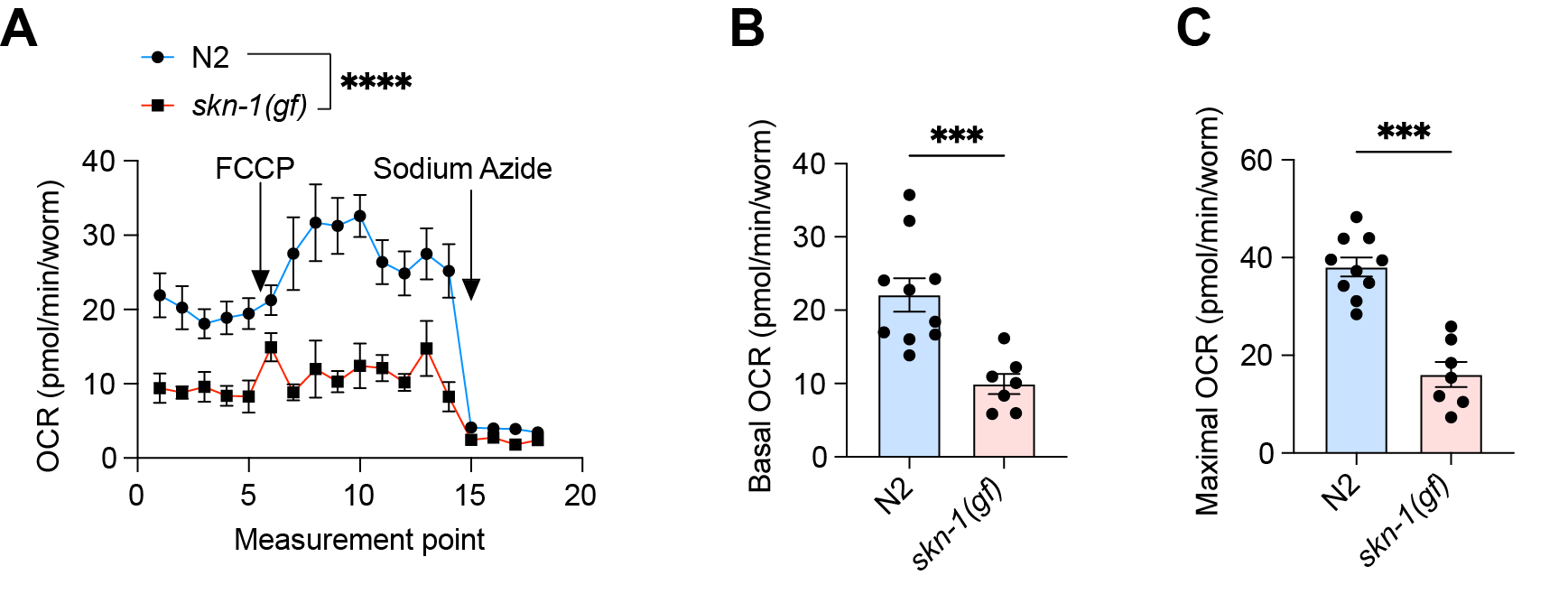


**Figure S9. Reduced oxygen consumption rates in *skn-1(gf*) mutants.**

(A) OCR measurements over time in wild-type (N2) and *skn-1(gf)* mutant worms. FCCP (an uncoupler) and sodium azide (a mitochondrial inhibitor) were sequentially injected at the indicated time points. (B) Quantification of basal OCR before FCCP injection. (C) Quantification of maximal OCR following FCCP treatment. Data are presented as mean ± SEM. Each dot represents one biological replicate consisting of a group of ~10 worms. Statistical significance was determined by an unpaired two-tailed t-test; ***p < 0.001, ****p < 0.0001.

**Supplementary methods**

**1. Cell counting kit-8 assay for evaluating cell viability**

WT MEF cells (1 × 10^4^ cells/ml) were seeded in 96-well plates. After 24 hours, the cells were exposed to 6-PPD/6-PPDQ or 0.5% DMSO alone for 24hours in a CO_2_ incubator (Thermo Fisher Scientific, USA, Heracell 240i). At the end of treatment, add 10 μL of Cell Counting Kit-8 (Dojindo, Japan, Cell Counting Kit-8) solution to each well of the plate for 2 hours in a CO_2_ incubator. The color intensity was measured at 540 nm using a microplate reader (Synergy HTX, BioTek, USA).

**2. High-Performance Liquid Chromatography (HPLC)**

HPLC was performed on a 1260 series HPLC system (Agilent Technologies, Inc., California, USA). The solvent used was DW and Acetonitrile, a gradient system was used at a flow rate of 0.5mL/min for analysis, and a Cosmosil C18 column (length, 150 mm; inner diameter, 4.6 mm; particle size, 5 µm; Milford, Massachusetts, USA) was used. The solvent conditions used in the mobile phases were 0-10 min at 10-15% B, 10-20 min at 20%, 20-30min at 25%, 30-40 min at 40%, 40-50 min at 70%, 50-60 min at 95%, and 60-70 min at 95%. The analysis was performed at a wavelength of 254 nm and a temperature of 35 ℃.

The High-Performance Liquid Chromatography (HPLC) analysis conditions:

| HPLC | | | | |
| --- | --- | --- | --- | --- |
| Instrument | Agilent 1260 series HPLC | | | |
| Column |  | | | |
| Flow rate | 0.5 mL/min | | | |
| Oven Temperature | 35 ˚C | | | |
| Detector | UV – Vis ( 254nm ) | | | |
| Mobile phase | A | DW | | |
|  | B | ACN | | |
|  | Gradient | min | A% | B% |
|  |  | 0 | 90 | 10 |
|  |  | 10 | 85 | 15 |
|  |  | 20 | 80 | 20 |
|  |  | 30 | 75 | 25 |
|  |  | 40 | 60 | 40 |
|  |  | 50 | 30 | 70 |
|  |  | 60 | 5 | 95 |
|  |  | 70 | 5 | 95 |
| 90 min A (5%) and B (95%) – wash time | | | | |

**3. Quantitative RT‑PCR (qPCR) analysis**

Total RNA was extracted using TRIzol, and 2 µg was reverse transcribed into cDNA in a 20 µl reaction using the SensiFAST cDNA Synthesis Kit (Bioline). qPCR was performed on a Thermal Cycler Dice® Real-Time System III (TaKaRa Bio, Japan) using the 2× SensiFAST SYBR Fluorescein Kit. Relative mRNA levels were calculated using the ΔΔCt method, with act-1 as the internal control. Each data point represents the average of at least three independent biological replicates. The primers used for qPCR are listed below.

| **Gene** | **Forward sequence** | **Reverse sequence** |
| --- | --- | --- |
| *gst-4* | GGACTCGCTGGAAAAACTGC | CGGGCTGGTTCAACAACTTC |
| *gst-5* | TGCCGGACAACAATACGAGG | AAGAAACGAGCAATCGCGTG |
| *gst-33* | TGCAGAGGCTTCGAGAGCTA | GTGGGATCTTCTCACCGTCG |
| *sod-3* | AAAGGAGCTGATGGACACTATTAAGC | AAGTTATCCAGGGAACCGAAGTC |
| *act-1* | ACGACGAGTCCGGCCCATCC | GAAAGCTGGTGGTGACGATGGTT |
