# Supplementary material for: 6-PPD induces mitochondrial dysfunction and reduces healthspan and lifespan through SKN-1 in Caenorhabditis elegans
Source: J Hazard Mater. Author manuscript; Available in PMC 2026 Jun 18. (PMC13277346; doi:10.1016/j.jhazmat.2025.140332)
Supplement: 2 [file NIHMS2185873-supplement-2.docx]

**Table S1. Summary of *C. elegans* lifespan assay data.**

| **Related figure** | **Genotype** | **Median lifespan** | **Number** | **P-value (vs. asterisked group)** |
| --- | --- | --- | --- | --- |
| Figure 4F | WT + DMSO* | 13 | 241 |  |
|  | WT + 6-PPD | 12 | 269 | <0.0001* |
| Figure 7A | WT + DMSO* | 14 | 86 |  |
|  | WT+ 0.5 mM 6-PPD** | 14 | 88 | <0.005* |
|  | *skn-1(zj15)* + DMSO*** | 11 | 167 | <0.0001* |
|  | *skn-1(zj15)* + 0.5 mM 6-PPD | 13 | 189 | <0.005**, <0.05***, |
| Figure 7K | WT + DMSO* | 14 | 75 |  |
|  | WT + 0.5 mM 6-PPD** | 11 | 82 | <0.001* |
|  | *daf-16(mu86)* + DMSO*** | 14 | 71 | <0.05* |
|  | *daf-16(mu86)* + 0.5 mM 6-PPD | 10 | 79 | NS**, <0.0001*** |
| Figure 8O | WT + DMSO* | 14.5 | 181 |  |
|  | WT+ 0.5 mM 6-PPD** | 14 | 141 | <0.05* |
|  | *skn-1(lax188)* + DMSO*** | 14 | 154 | <0.001* |
|  | *skn-1(lax188)* + 0.5 mM 6-PPD | 12 | 161 | <0.05***, <0.0001** |
| Figure S5A | WT+ DMSO* | 11 | 77 |  |
|  | WT+ 6-PPD** | 10 | 85 | <0.05* |
|  | WT +6-PPDQ | 9 | 30 | <0.05**, <0.001* |
| Figure S7A | *skn-1(zj15)* + DMSO* | 11 | 92 |  |
|  | *skn-1(zj15)* + 0. 1 mM 6-PPD** | 11 | 99 | <0.05* |
|  | *skn-1(zj15)* + 0.5 mM 6-PPD*** | 13 | 99 | <0.0001* |
|  | *skn-1(zj15)* + 1 mM 6-PPD | 13 | 92 | <0.0001* |
